# Modulation of microbiome diversity and cytokine expression is influenced in a sex-dependent manner during aging

**DOI:** 10.3389/frmbi.2022.994464

**Published:** 2022-10-10

**Authors:** Sarah E. Webster, Duncan Vos, Thomas L. Rothstein, Nichol E. Holodick

**Affiliations:** ^1^ Center for Immunobiology, Department of Investigative Medicine, Western Michigan University Homer Stryker M.D. School of Medicine, Kalamazoo, MI, United States; ^2^ Division of Epidemiology and Biostatics, Department of Biomedical Sciences, Western Michigan University Homer Stryker M.D. School of Medicine, Kalamazoo, MI, United States

**Keywords:** microbiome, aging, sex, immune system, B-1 cells

## Abstract

The microbiome and immune system have a unique interplay, which influences homeostasis within the organism. Both the microbiome and immune system play important roles in health and diseases of the aged including development of cancer, autoimmune disorders, and susceptibility to infection. Various groups have demonstrated divergent changes in the gut microbiota during aging, yet the compounding factor of biological sex within the context of aging remains incompletely understood, and little is known about the effect of housing location in the composition of gut microbiota in the context of both sex and age. To better understand the roles of sex, aging, and location in influencing the gut microbiome, we obtained normal healthy BALB/cByJ mice from a single source and aged male and female mice in two different geographical locations. The 16S rRNA was analyzed from fecal samples of these mice and cytokine levels were measured from serum. 16S rRNA microbiome analysis indicated that both age and sex play a role in microbiome composition, whereas location plays a lesser role in the diversity present. Interestingly, microbiome changes occurred with alterations in serum expression of several different cytokines including IL-10 and IL-6, which were also both differentially regulated in context to sex and aging. We found both IL-10 and IL-6 play a role in the constitutive expression of pSTAT-3 in CD5+ B-1 cells, which are known to regulate the microbiome. Additionally, significant correlations were found between cytokine expression and significantly abundant microbes. Based on these results, we conclude aging mice undergo sex-associated alterations in the gut microbiome and have a distinct cytokine profile. Further, there is significant interplay between B-1 cells and the microbiome which is influenced by aging in a sex-dependent manner. Together, these results illustrate the complex interrelationship among sex, aging, immunity, housing location, and the gut microbiome.

## 1 Introduction

Both the microbiome and immune system change with advancing age. Age-related changes to the microbiome include decrease in microbial diversity, increase in pathobionts, and increased gut permeability (Falony et al., 2016; Elderman et al., 2018). Changes in the composition of the microbiome are associated with the development of obesity and related metabolic disorders (Ley, 2010; Tilg and Kaser, 2011), maintenance of gut barrier integrity (Turner, 2009; Jandhyala et al., 2015; Gwak and Chang, 2021), intestinal pro- and anti-inflammatory balance (Desai et al., 2016; Bolte et al., 2021), immune and cardio-metabolic health (Hansen et al., 2015; López et al., 2016; Sanchez-Alcoholado et al., 2017), and homeostasis of the gut-brain axis (Rhee et al., 2009; Cryan and O’Mahony, 2011; Lyte, 2013). To date, a large body of evidence has shown that older people maintain a compositionally distinct microbiota as compared with younger adults; these changes are associated with gradients in clinical frailty and inflammatory status (O’Toole and Jeffery, 2018). Alterations in the composition and function of the microbiome have been attributed to several factors including: senescence, changed lifestyle and dietary schedules, geographic location, lesser mobility, reduced intestinal function, altered gut morphology, recurrent infections, and weakened immune system strength (Langille et al., 2014; Shibagaki et al., 2017; Bodogai et al., 2018; Nagpal et al., 2018). Age-related changes to the immune system include skewed hematopoiesis, reduced lymphopoiesis, impaired innate and adaptive immune responses, impaired memory response, increase in autoimmune disease, and chronic inflammation, all of which may be associated with microbiome dysbiosis (Kelly and Mulder, 2012; Levy et al., 2017; Bana and Cabreiro, 2019; DeJong et al., 2020; Bosco and Noti, 2021). Since the microbiome regulates the host-immune system and the immune system regulates the microbiome, each can play inter-dependent and independent roles in such age-related changes contributing to various diseases of the aged (Mcdermott and Huffnagle, 2014; Neish, 2014; Thaiss et al., 2016; Hagan et al., 2019).

Recent studies have demonstrated the important relationship between the microbiome and B cells in health and disease. The microbiota can influence early B cell development, B cell repertoire diversification, IgA production, IgE production, B cell metabolism, and activation and differentiation of B cells (Round and Mazmanian, 2009; Brown et al., 2013; Belkaid and Hand, 2014; Petta et al., 2018; Dominguez-Bello et al., 2019; Baumgarth, 2021). Interestingly, commensal bacteria contain orthologues to a common systemic lupus erythematosus (SLE) autoantigen, and exposure to such bacterial orthologues can prime production of autoantibodies (Silverman, 2019). Translocation of commensal bacteria has also been shown to lead to a SLE-like disease *via* an increase in IFN-gamma and subsequent autoantibody production (López et al., 2016). Gut dysbiosis and altered tryptophan metabolism have also been shown to contribute to autoimmunity in lupus-prone mice (Choi et al., 2020), which carry the Sle2c1 locus leading to CD5+ B-1 cell expansion (Potula et al., 2012). Furthermore, commensal bacteria have been shown to activate monocytes leading to the conversion of CD5+ B-1 cells to 4-1BBL+ CD5+ B-1 cells, resulting in impaired insulin signaling (Bodogai et al., 2018). B-1 cells produce 80-90% of the natural IgM (Förster and Rajewsky, 1987; Lalor et al., 1989; Blandino and Baumgarth, 2019), which is present in serum in the absence of infection or intentional immunization and is required for many immune system functions (Boes et al., 1998; Ehrenstein et al., 2000; Binder and Silverman, 2005; Haas et al., 2005; Weksler et al., 2009; Ehrenstein and Notley, 2010; Kearney et al., 2015; Nguyen et al., 2015; Tsiantoulas et al., 2017). We recently demonstrated sex influences age-related changes in natural IgM and natural antibody producing CD5+ B-1 cells (Webster et al., 2022).

Importantly, the microbiome is an inherent factor shown to manifest sex-related differences; sex hormones have been shown to influence the microbiota composition and microbes can affect levels of sex hormones (Markle et al., 2013; Jašarević et al., 2016; Elderman et al., 2018; Kaliannan et al., 2018; Kaur et al., 2021). Recently, it was found that disruption of the maternal-offspring transmission of microbes during important periods of development leads to long-term offspring health consequences with males more susceptible to these effects (Jašarević et al., 2021). Differences in the microbiota between sexes have also shown to play a role in response to vaccination (Fischinger et al., 2019). Additionally, it was shown in a model of type 1 diabetes that the gut microbiota alters the levels of sex hormones in mice and plays a role in regulation of autoimmune disease fate (Markle et al., 2013).

Given the changes of the gut microbiome demonstrated with age and sex separately, we sought to investigate linkages specifically between aging, sex, the microbiome, and the immune system at the same time in a controlled system. There are many confounding factors in human studies, including changes in diet, medications, housing status, and physical location that cannot be easily controlled, making it difficult to identify direct effects of sex- and age-related changes to the microbiome. In this study, we obtained healthy BALB/c-ByJ mice from a single source and aged male and female mice in two different locations. 16S rRNA microbiome analysis indicated age and sex play a large role in microbiota composition; additionally, the housing location of the mice influenced the composition of the gut microbiome. Mucosal IgA, IL-10, and IL-6 regulate the microbiome (López et al., 2016; Burrello et al., 2018; He et al., 2019; Koelman et al., 2019; Luckett-Chastain et al., 2021) and are produced by CD5+ B-1 cells (Kroese et al., 1989; O’garra et al., 1992; O’garra and Howard, 1992; Kroese and Bos, 1999; Macpherson et al., 2000). We have recently demonstrated aged male and female mice have significantly more serum IgA, whereas only aged female mice have increased numbers of CD5+ B-1 cells and serum IL-5 as compared to aged males (Webster et al., 2022), which is required for maintenance and antibody secretion of CD5+ B-1 cells (Moon et al., 2004). Considering the differential changes we observed in the microbiome between young and aged male and female mice, we investigated whether there may be differences in serum cytokine levels in these mice. Interestingly, we observed changes in serum expression of several different cytokines including IFNγ, IL-1β, IL-10, and IL-6, which were altered in association with sex and age. We then asked whether IL-10 and/or IL-6 could affect CD5+ B-1 cell signaling. We found both IL-10 and IL-6 play a role in the constitutive expression of pSTAT-3 in CD5+ B-1 cells. Together these results demonstrate sex and age affect composition of the gut microbiome and serum cytokine expression, which can affect CD5+ B-1 cells.

## 2 Materials and methods

### 2.1 Animals and sample collection

BALB/c-ByJ mice were purchased from The Jackson Laboratory (#001026; Bar Harbor, ME) at 6 weeks of age and were aged to either 2-3-months or 17-26-months of age in one of two separate animal facilities: the Feinstein Institute for Medical Research (FIMR, Manhasset, NY) or Western Michigan University Homer Stryker M.D. School of Medicine (WMed, Kalamazoo, MI). All animals were housed in identical environments with 5 mice per cage with a 12-hour light/12-hour dark cycle and ad libitum to water and food. Specifically, mice housed at FIMR were fed LabDiet 5053 and the mice housed at WMed were fed LabDiet 5002, which are nearly identical certified rodent diets (**Table S1**); chemical, mineral, and vitamin composition are identical though 5002 contains both soybean oil and ground soybean hulls while 5053 only contains soybean oil. Additionally, the mice were housed in identical bedding (irradiated ¼ inch bed-o-cob) and cages (Allentown micro-isolator cages). Mice were cared for and handled in accordance with the Guide for the Care and Use of Laboratory Animals, National Institutes of Health, and institutional guidelines. All studies were approved by each institutional IACUC.

Fecal samples from young and aged mice were collected at the same time by clean catch and into a 1.5mL sterile collection tube before being placed on dry ice for shipment. After fecal collection, mice were euthanized by CO_2_ overdose followed by bilateral pneumothorax. Serum was collected at the time of euthanasia from all mice; however, only serum samples from the mice at WMed were available for cytokine analysis. Fecal samples from the FIMR were shipped on dry ice to Second Genome for analysis. Fecal samples from WMed were shipped on dry ice to Veracet (an off shoot of Second Genome) for analysis.

### 2.2 16S rRNA whole microbiome sequencing

#### 2.2.1 Sample isolation

Location/Batch one samples (FIMR) were run by Second Genome in which the nucleic acid isolation was performed with MoBio PowerMag Microbiome Kit (Carlsbad, CA) according to manufacturer’s guidelines and optimized for high-throughput processing. Location/Batch two samples (WMed) were run by Veracet in which nucleic acid was isolated with the Qiagen MagAttract PowerMicrobiome DNA/RNA Kit according to manufacturer’s guidelines and optimized for high-throughput processing. The MagAttract PowerMicrobiome DNA/RNA Kit was formerly sold by MoBio as PowerMag Microbiome Kit. All samples were quantified *via* the Quibit Quant-iT dsDNA High Sensitivity Kit (Invitrogen, Life Technologies, Grand Island, NY) to ensure samples met minimum concentration and mass of DNA.

#### 2.2.2 Library preparation

To enrich the sample for bacterial 16S V4 rDNA region, DNA was amplified using fusion primers designed against the surrounded conserved regions and were tailed with sequences to incorporate adapters and indexing barcodes (Illumina, San Diego, CA). The primer sequences (not including the barcode, linker, and pad sequences) used at Second Genome and Veracet are as follows: FWD:GTGYCAGCMGCCGCGGTAA; REV:GGACTACNVGGGTWTCTAAT. The PCR is the library prep step since the primers include barcodes, etc. Each sample was PCR amplified with two differently bar coded V4 fusion primers and PCR products were quantified by fluorometric method (Qubit or PicoGreen; Invitrogen, Life Technologies, Grand Island, NY). Samples that met post-PCR quantification minimum were pooled in an equimolar fashion and advanced for sequencing.

#### 2.2.3 Profiling method

A pool containing 16S V4 enriched, amplified, barcoded samples were loaded into a MiSeq reagent cartridge, and then onto the instrument along with the flow cell. After cluster formation on the MiSeq instrument, the amplicons were sequenced for 250 cycles with custom primers designed for paired-end sequencing.

### 2.3 16S rRNA data analysis methods

#### 2.3.1 Overview

The full data analysis pipeline for Veracet’s Microbial Profiling Service incorporates several separate stages: pre-processing, summarization, normalization, alpha diversity metrics, beta diversity metrics, ordination/clustering, sample classification, and significance testing. Second Genome’s analysis software package was used for statistical analysis.

#### 2.3.2 OTU selection

Sequenced paired-end reads were merged using USEARCH (Edgar, 2010) and the resulting sequences were compared to a Veracet strain database using USEARCH. All sequences hitting a unique strain with an identity >99% were assigned a strain OTU. To ensure specificity of the strain hits, a difference of >0.25% between the identity of the best hit and the second-best hit was required. For each strain OTU, one of the match reads was selected as representative and all sequences were mapped by USEARCH against the strain OTU representatives to calculate strain abundances. The remaining non-strain sequences were quality filtered and dereplicated with USEARCH. Resulting unique sequences were then clustered at 97% by UPARSE (*de novo* OTU clustering (Edgar, 2013);) and a representative consensus sequence per *de novo* OTU was determined. The UPARSE clustering algorithm comprises a chimera filtering and discards likely chimeric OTUs. All non-strain sequences that passed the quality filtering were mapped to the representative consensus sequences to generate an abundance table for *de novo* OTUs. Representative OTU sequences were assigned taxonomic classification *via* mothur’s Bayesian classifier, trained against the Greengenes reference database (McDonald et al., 2012) of the 16S rRNA gene sequences clustered at 99%.

#### 2.3.3 Summarization

After the taxa are identified for inclusion in the analysis, the values used for each taxa-sample intersection are populated with the abundance of reads assigned to each OTU in an “OTU table”. A corresponding table of OTU Greengenes classification is generated as well.

#### 2.3.4 Diversity metrics

Alpha diversity is a measure of richness identified as the sum of unique OTUs found in each sample. Shannon diversity utilizes the richness of a sample along with the relative abundance of the present OTUs to calculate a diversity index. Significance between sample alpha-diversity metrics was determined using Chi-squared and Kruskal-Wallis.

Beta diversity was calculated by inter-comparing profiles in a pair-wise fashion to determine a dissimilarity score and store it in a distance dissimilarity matrix. The dissimilarity score produced by the distance function was used to compare samples with low dissimilarity scores and high dissimilarity scores, corresponding to similar samples and different samples, respectively. Abundance-weighted sample pair-wise differences were calculated using the Bray-Curtis dissimilarity as the ratio of the summed absolute differences in counts to the sum of abundances in the two samples. The binary dissimilarity values were calculated with the Jaccard index to compare the number of mismatches (OTUs present in one but absent in the other) in two samples relative to the number of OTUs present in at least one of the samples.

#### 2.3.5 Ordination, clustering, and classification methods

Two-dimensional ordinations and hierarchical clustering maps of the samples in the form of dendrograms were created to graphically summarize the inter-sample relationships across age, sex, and location. Principal Coordinate Analysis (PCoA) is a method of two-dimensional ordination plotting that was used to help visualize complex relationships between samples. PCoA uses the sample-to-sample dissimilarity values to position the points relative to each other by maximizing the linear correlation between the dissimilarity values and the plot distances. Dendrograms were created using the Ward method of linkage for hierarchically clustering to evaluate hierarchical relationships between samples.

#### 2.3.6 Whole microbiome significance testing

Permutational Analysis of Variance (PERMANOVA) was utilized to determine dissimilarity measures. Veracet used a Conditional Monte Carlo (CMC) permutation test. The CMC test gives similar results to carrying out all possible permutations. In this randomization/Monte Carlo permutation test, the samples are randomly reassigned to the various sample categories, and the between-category differences are compared to the true between-category differences. The PERMANOVA utilized the sample-to-sample distance matrix directly, not a derived ordination or clustering outcome.

#### 2.3.7 Taxon significance testing

Univariate differential abundance of OTUs was evaluated using a negative binomial noise model for the overdispersion and Poisson process intrinsic to this data, as implemented in the DESeq2 package and described for microbiome applications (Love et al., 2014). This method accounts for both technical and biological variability between experimental conditions. Univariate differential abundance of OTUs is tested using a negative binomial noise model for the overdispersion and Poisson process intrinsic to this data, as implemented in the DESeq2 package and described for microbiome applications. It takes into account both technical and biological variability between experimental conditions. DESeq was run under default settings and q-values were calculated with the Benjamini-Hochberg procedure to correct *p*-values, controlling for false discovery rates. Features were only considered signifanct if their FDR-corrected *p*-value was less than or equal to 0.05 per Veracet.

### 2.4 Pro-inflammatory and cytokine analysis

#### 2.4.1 Serum collection

Whole blood was collected from each individual BALB/c-ByJ naïve mouse at the time of the euthanasia at indicated age. Whole blood was allowed to clot at room temperature for 20 minutes before centrifugation and the supernatant (serum) was then collected. The serum was stored at -20°C until use.

#### 2.4.2 V-PLEX mouse cytokine 19-plex panel

Nineteen plasma cytokines (IFNγ, IL-1b, IL-2, IL-4, IL-5, IL-6, IL-9, KC/GRO, IL-10, IL-12p70, IL-15, IL-17A/F, IL-27p28/IL-30, IL-33, IP-10, MCP-1, MIP-1a, MIP-2, TNF-a) were measured using chemiluminescence-based assays from Meso Scale Discovery (MSD; Gaithersburg, MD) using the V-PLEX 19 mouse cytokine 19-plex panel (MSD; catalog #K15255D-2). The detection ranges are IFNγ: 0.04–570 pg/mL IL-1β: 0.11–1030 pg/mL; IL-2: 0.22–1570 pg/mL; IL-4: 0.14–1060 pg/mL; IL-5: 0.07–590 pg/mL; IL-6: 0.61–3140 pg/mL; KC/GRO: 0.24–1230 pg/mL; IL-9: 3.84 – 2,600 pg/mL; IL-10: 0.95–2030 pg/mL; IL-12p70: 9.95–20 600 pg/mL; IL-15: 16.0 - 26,000 pg/mL; IL-17A/F: 0.231 - 1,620 pg/mL; IL-33: 0.364 – 1,950 pg/mL; IL-27p28/IL-30: 1.39 – 6,500 pg/mL; IP-10: 0.328 - 650 pg/mL; MCP-1: 0.672 – 325 pg/mL; MIP-1α: 0.081 - 390 pg/mL; MIP-2: 0.053 - 423 pg/mL; TNF-α: 0.13–403 pg/mL. All assays were performed in duplicate with at least n=5 animals for each age/sex group. Cytokine analysis was performed on serum samples obtained from the same WMed cohort of mice as the microbiome analysis. Serum IgA and IL-5 concentrations were obtained from WMed mice and performed in a previously published study (Webster et al., 2022), which were of the same cohort of mice used for microbiome analysis. Analyses were done using a QuickPlex SQ 120 instrument (MSD), DISCOVERY WORKBENCH 5.1 software (MSD), and GraphPad Prism. Results are shown for cytokine samples with a coefficient of variation (CV) <12 (IFNγ, IL-1β, IL-10, IL-6, IL-5, KC/GRO); therefore, results for cytokines with a high CV were not included. All samples were run at the same time.

### 2.5 Statistical and correlation analysis

Means and standard errors were calculated for the cytokine expression data using GraphPad Prism. To test significance, a two-way ANOVA was run with Tukey’s multiple comparison test. Correlation analysis was performed to evaluate the relationship between strain abundance (using data from WMed & FMIR) and cytokine expression (using serum from WMed) for strains that significantly differed in relative abundance by age and sex. Non-linear Spearman Rank-order correlation was used to evaluate the strength of the correlation between strain abundance and cytokine expression with GraphPad Prism, calculating a Spearman correlation value for each strain and cytokine relationship (r value). The *p*-value associated with the Spearman Rank-order correlation was calculated within Prism (two-tailed). The resulting r values were then heat mapped against the logarithm base 10 of relative abundance (used to minimize the variation) using Morpheus matrix visualization and analysis software (Morpheus, https://software.broadinstitute.org/morpheus). Scatter plots were provided for all significant correlations (*p*<0.05 and α<0.05). Specific significance tests are listed on each figure legend.

### 2.6 Analysis of IL-6 and IL-10 on CD5+ B-1 cell signaling

#### 2.6.1 Isolation of CD5+ B-1 cells

Peritoneal lavage and spleen removals were performed on euthanized BALB/c-ByJ male mice. Spleens were homogenized using the frosted ends of microscope slides and then passed through a 70-um cell strainer. All samples were treated with RBC lysis buffer for 2 minutes (Lonza), subsequently diluted with HBSS with 2.5% FBS, and then centrifuged at 1200rpm for 10 minutes. The cells were resuspended in HBSS with 2.5% FBS, stained with immunofluorescent antibodies, and then sorted on an Influx cell sorter (BD Biosciences) with gating on live cells by forward side scatter. The following antibodies were obtained from BD Pharmingen: CD19 (clone ID3), B220/CD45 (clone RA3-6B2), and CD5 (clone 53-7.3).

#### 2.6.2 pSTAT-3 detection in CD5+ B-1 cells after IL-6 and/or IL-10 neutralization

Isolated CD5+ B-1 cells were treated with 10 μg/ml of anti-IL-6 (R&D Systems AF-406-NA) and/or anti-IL-10 (R&D Systems AB-417-NA) neutralizing antibody for 24 hours at 37°C. As a control for the effectiveness of each neutralizing antibody, B2 cells were stimulated with IL-6 and/or IL-10 (10 ng/ml) (R&D Systems) for 24 hours in the presence or absence of 10 ug/ml anti-IL-6 and/or anti- IL-10 (R&D Systems). In addition, we utilized a goat IgG antibody as a control for neutralization (R&D Systems). Afterwards, cells were pelleted, frozen, and lysed in NP-40 lysis buffer for western blot analysis of phosphorylated STAT3. Total protein was resolved on a 10% SDS-PAGE gel at 80 constant volts for about 2 hours. A protein standard was used to determine protein size (BioRad #1610374). A sandwich was prepared in between two sponges using two pieces of chromatography paper (Whatman #3030 917), the SDS PAGE gel, nitrocellulose pre-soaked in distilled water for 10 minutes, and then two more pieces of chromatography paper were stacked on top of the nitrocellulose, in that order. The separated proteins were then transferred to Hybond ECL nitrocellulose membrane (Amersham #RPN303D) at 100 volts for one hour using the Mini Trans-Blot Electrophoretic Transfer Cell (BioRad #170-3930). The membrane was then blocked with 5% non-fat dry milk in TBST (TBS, Tris Base, NaCl, 0.1% tween, pH 7.6) for one hour at room temperature with gentle rocking or rotating. The membrane was incubated with one of the antibodies, either Phospho-STAT3 (Ty705, Cell Signaling 9138) or STAT3 (Cell Signaling 9139) in 2% non-fat dry milk overnight at 4°C. Afterwards, the membrane was washed with Tris-buffered saline with 0.1% Tween-20 (TBST) once for 15 minutes and then three 5-minute washes. Next, the blot was probed with goat anti-mouse-IgG-HRP (Santa Cruz Biotechnology #sc-2005) for one hour at room temperature in 2% non-fat dry milk in TBST. Again, the membrane was washed with TBST once for 15 minutes and then three 5-minute washes. Detection was performed with enhanced chemiluminescence (Pierce #34080) and then developed with autoradiography film (Denville #E3012).

#### 2.6.3 pSTAT-3 detection in CD5+ B-1 cells obtained from IL-6 or IL-10 knockout mice

IL-10 knockout (B6.129P2-Il10^tm1Cgn^/J, strain #002251) and IL-6 knockout (B6.129S2-Il6^tm1Kopf^/J, strain #002650) mice of 6–8 weeks age were obtained from The Jackson Laboratory (Bar Harbor, ME). Peritoneal lavage and spleen removals were performed on euthanized IL-10 and IL-6 knockout male mice. Spleens were homogenized using the frosted ends of microscope slides and then passed through a 70-um cell strainer. All samples were treated with RBC lysis buffer for 2 minutes (Lonza), subsequently diluted with HBSS with 2.5% FBS, and then centrifuged at 1200rpm for 10 minutes. The cells were resuspended in HBSS with 2.5% FBS, stained with immunofluorescent antibodies, and then sorted on an Influx cell sorter (BD Biosciences) with gating on live cells by forward side scatter. The following antibodies were obtained from BD Pharmingen: CD19 (clone ID3), B220/CD45 (clone RA3-6B2), and CD5 (clone 53-7.3). Isolated CD5+ B-1 cells were treated were pelleted, frozen, and lysed in NP-40 lysis buffer for western blot analysis of phosphorylated STAT3.

## 3 Results

### 3.1 Sample collection and sequencing of the 16S rRNA V4 region

**Figure 1:**
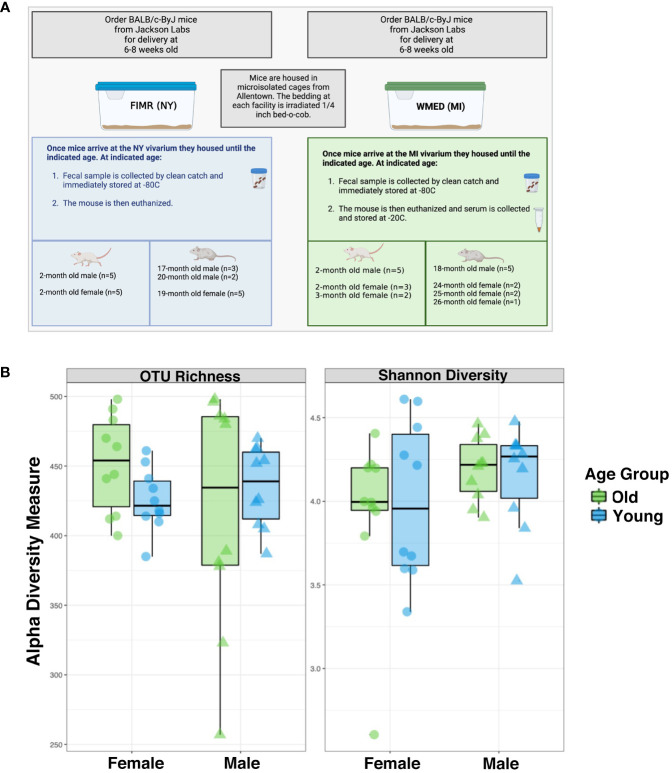
Alpha diversity estimate between groups show no significant differences between males and females for whole microbiome analysis. **(A)** Experimental design. Samples from BALB/c-ByJ mice were collected at two different locations: The Feinstein Institute for Medical Research (FIMR) and Western Michigan University Homer Stryker M.D. School of Medicine (WMed). Mice were purchased from Jackson Laboratory at 6 weeks of age and then housed in the respective animal facility until the aged indicated. **(B)** Alpha diversity was analyzed by two means: OTU richness and the Shannon diversity index. OTU richness represents the number of OTUs present in each sample. Young mice are blue and old mice are green. The female mice had an OTU richness of 434 ± 65.3 while the male mice had an OTU richness of 430 ± 25.6 (*p*=0.3). The Shannon diversity index considers the richness and evenness of OTUs within a sample and was calculated to be 3.97 ± 0.475 for female mice and 4.17 ± 0.24 for male mice (*p*=0.77). Statistics used = Kruskal-Wallis. Figure 1A was generated using BioRender.

A total of 40 healthy BALB/c-ByJ mice of two age groups; 10 females and 10 males between 2-3 months old (young group) and 10 females and 10 males between 17-26 months old (old group), were used for this study. The mice were housed and aged in two different locations (also referred to as batches) (**Figure 1A**). The two locations were The Feinstein Institute for Medical Research (FMIR) in Manhasset, NY (batch 1) and Western Michigan University Homer Stryker M.D. Medical School (WMed) in Kalamazoo, MI (batch 2). The bacterial community of the gut was analyzed from fecal collection from all animals (see Methods). The bacterial 16S rRNA gene V4 region (16S) was sequenced using Illumnia MiSeq to obtain a total of 12,231,644 total quality-filtered reads. For the 40 samples that were analyzed, the total number of reads per sample from each age and sex group was greater than 50,000.

Clustering of the high-quality 16S reads generated a total of 1,136 operational taxonomic units (OTUs) from all samples after removal of OTUs unclassified at the kingdom level; removal of spurious OTUs was completed by independent filtering with OTUs seen at least once within 5% of the data set being kept, reducing the number of OTUs to 842 (**Supplementary Figure S1**). Importantly, rarefaction curves for most samples approached saturation indicating that communities were sufficiently sampled to characterize the microbiome (**Supplementary Figure S1**). These data indicate that the sample size of the gut microbiome was sufficient to enumerate and cover the OTUs shared within the sequencing profiles. Within the sample populations, 100% of the kingdom taxa were identified, 99.93% of phylum taxa were identified, and 63.14% of the genus taxa were identified (**Supplementary Figure S2**).

### 3.2 No significant differences in alpha diversity of the microbiota between male and female mice of any age

First, the alpha diversity, or species richness, was analyzed based on the observed OTU number and the Shannon index (**Figure 1B**). These two metric analyses gave similar results, revealing the alpha diversity of the male microbiome was like that of the female microbiome; there were no significant differences in alpha diversity based on age. In females the average OTU richness was 439 ± 31.5 (SD) while the average males were 426 ± 62.1 (SD), with the Shannon diversity having an average of 3.97 ± 0.475 (SD) and 4.17 ± 0.24 (SD) respectively. This indicates that alpha diversity does not significantly differ by sex or age. We have summarized these findings in **Supplementary Table 2**. As there was no change in the alpha diversity driven by sex-dependent aging, we next examined changes to the beta diversity in these groups.

### 3.3 Differences in beta diversity in gut microbiome between age and sex groups

**Figure 2:**
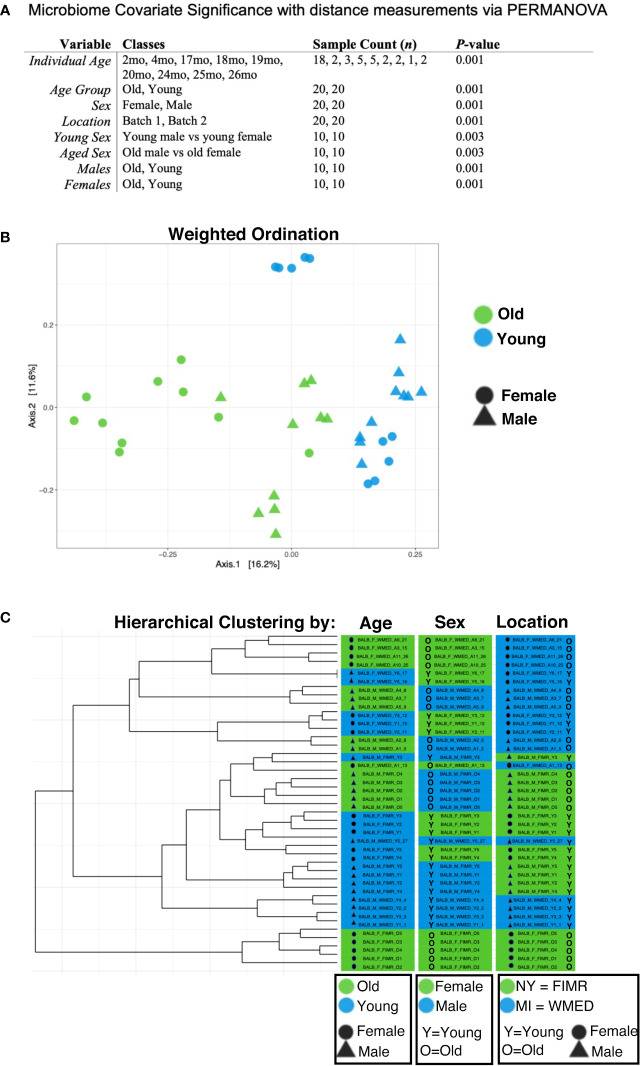
Beta diversity analysis shows significant contributions from age, sex, and location on microbiota. **(A)** Microbiome covariate significance was measured with distance measurements *via* PERMANOVA. Each variable tested is listed with relative classes, n for each class, and calculated *p*-value. **(B)** Weighted ordination by dimensional reduction of the Bray-Curtis dissimilarity between microbiome samples, using the PCoA ordination method shows that samples separated by age group (*p*=0.001) and sex *(p*=0.001). To further examine these relationships, samples were clustered by the Ward’s method and Bray-Curtis distance. **(C)** Hierarchical clustering partially separated by age, sex, and location. A PERMANOVA using distance matrices was performed for each variable of interest to determine if they significantly contributed to the beta diversity of the samples. All variables tested had a statistical significance of *p* < 0.01 when tested separately.

The overall structural similarly and variation (beta diversity between samples) between the microbiomes from the age and sex groups were then examined using the Bray-Curtis dissimilarity distance analysis with Principal Coordinates Analysis (PCoA). Assessment of these differences by the Permutational Multivariate Analysis of Variance (PERMANOVA) showed that age group, sex, and location (batch) all significantly contributed to the beta diversity of the samples (**Figure 2A**). Interestingly, Bray-Curtis weighted ordination showed that the microbiome samples tended to cluster according to age and to a lesser degree by sex (**Figure 2B**), while unweighted ordination clustered according to location (batch) (**Supplementary Figure S2**). As samples separated by location on unweighted ordination, this indicates that there are substantial differences in the number and presence of OTUs detected between samples. However, this difference was not strongly observed in the weighted ordination which considers relative abundance of OTUs as well as presence/absence.

Hierarchical clustering demonstrates these data partially separated by age, sex, and location (**Figure 2C**). These data confirm previous reports of age-related alterations of the gut microbiome (Langille et al., 2014; Nagpal et al., 2018; Bana and Cabreiro, 2019; DeJong et al., 2020; Bosco and Noti, 2021) and demonstrate for the first time that sex-related alterations also play a role in the relative abundance and community species membership of the gut microbiome in the context of aging.

### 3.4 Taxonomic profiles of gut microbiomes differ between the age and sex groups and are influenced by location

**Figure 3:**
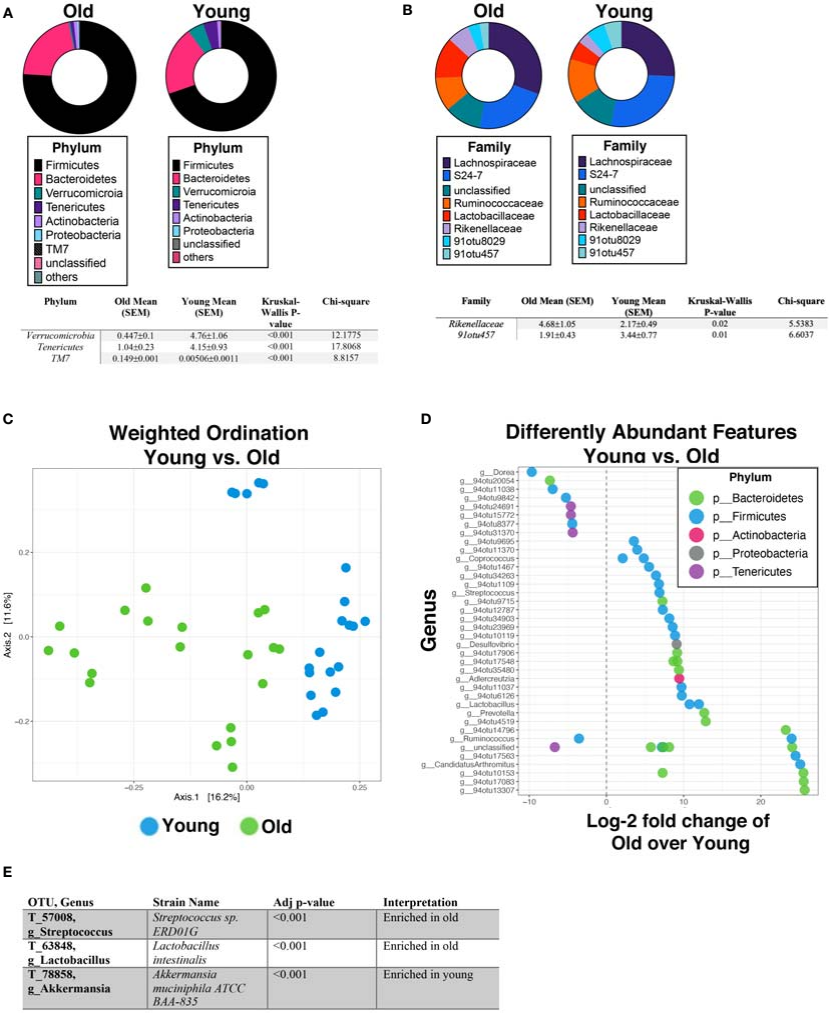
Age significantly impacts gut microbiome abundance and diversity. Percent abundance as means with SEM for the top eight phylum **(A)** and families **(B)** expressed between all old mice (17-26 months) vs all young mice (2-3 months). The two OTUs listed (91otu8029 and 91otu457) belong to the Clostridiales Family. Significant changes (*p*<0.05) are indicated the in the tables below each plot. **(C)** Weighted ordination by abundance indicates that samples cluster by age and partially by sex. **(D)** Top 50 significantly differently expressed OTUs between all aged mice vs young mice by phylum are represented out of a total of 170 significantly different features. **(E)** The feature selection summary for 6 OTUs identified at the strain level. Significance was tested using a negative binominal noise model with Poisson process corrected for FDR with Benjamini-Hochberg procedure.

Since the beta diversity analysis indicated clustering primarily between age and sex, we examined specific comparisons between age and sex groups. First, a comparison between all old and young mice was performed (**Figure 3A**). The differential expression of the microbial diversity was apparent between these two groups. The comparison of the proportional abundance of all old mice versus all young mice indicated that the TM7 phylum was significantly (*p<0.001*; Kruskal-Wallis) more abundant in old mice (0.149 ± 0.082%) than young mice (0.000482 ± 0.00026%), while Verrucomicrobia (4.76 ± 1.0644% vs 0.447 ± 0.100%; *p<*0.001) and Tenericutes (4.15 ± 0.928% vs 1.04 ± 0.2326%; *p<*0.001) were more abundant in young mice compared to old mice, respectively (**Figure 3A**). At the family level, significant differences were observed in *Rikenellaceae*, in which the aged population had higher abundance (4.68±1.05% vs 2.17±0.49%; *p<*0.05) and *Clostridiales* family 91otu457, in which the young had higher abundance (1.91±0.43% vs 3.44±0.77%; *p<*0.01*)* (**Figure 3B**). The first two ordination axes using the PCoA for the weighted ordination across age and sex accounted for 27.9% of sample variation (**Figure 3C**). Further analysis between the OTUs in the old and young populations showed that there were 170 significantly different features detected out of 842 tested, with a log_2_-fold change greater than 1 and a Benjamini-Hochberg corrected *p<*0.05 (**Figure 3D**). In old mice, the top enriched features were from *Candidatus*, 94otu17563, 94otu13307, and 94otu10153 genera while young mice were enriched in *Dorea*, 94otu11038, and 94otu20054 genera. Specifically, at the strain level, *Streptococcus* sp. and *Lactobacillus intestinalis* were enriched in the old group (*p=4.37e^-6^* and *p=*1.06e^-5^) while *Akkermansia muciniphila* (*p=*3.60e^-4^) was enriched in the young population (**Figure 3E**).

**Figure 4:**
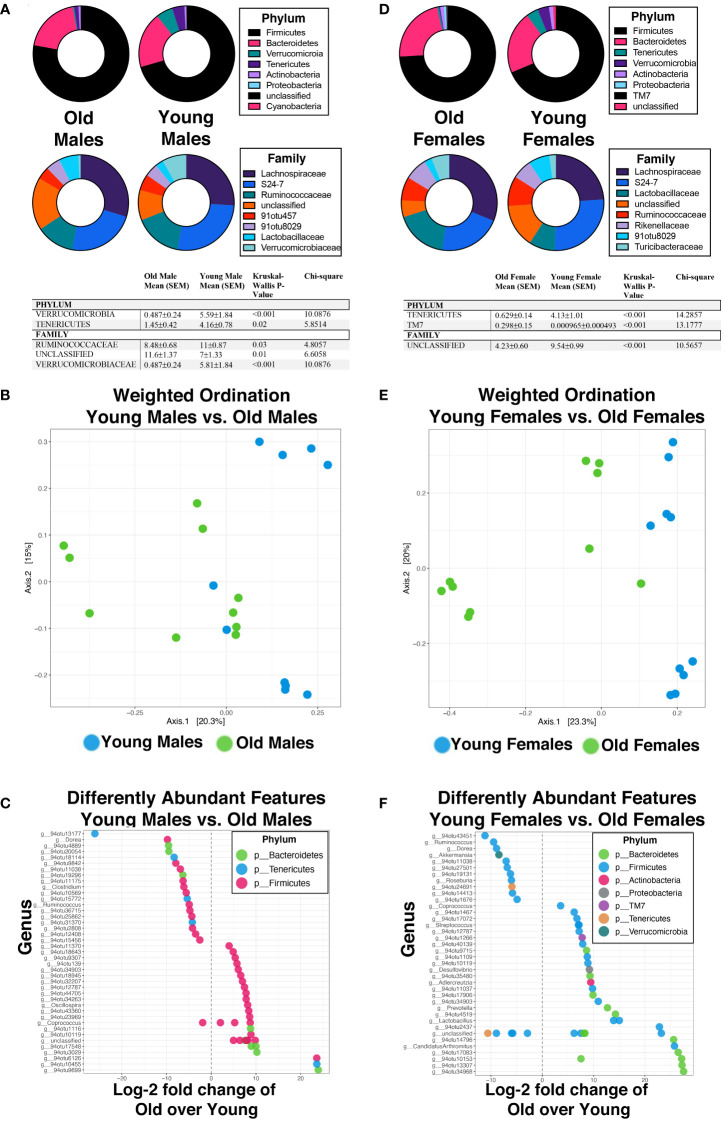
Aging in a sex-independent manner significantly changes the diversity and abundance of the gut microbiome in mice. **(A, D)** Percent abundance as means with SEM for the top eight phylum and families expressed between old and young male **(A)** and female **(D)** mice. The two OTUs listed (91otu8029 and 91otu457) belong to the Clostridiales Family. Significant changes (*p*<0.05) are indicated the in the tables. **(B, E)** Weighted ordination by abundance indicates that samples cluster by age for males **(B)** and by age for females **(E)**. **(C, F)** Top 50 significantly differently expressed OTUs. Significance was tested using a negative binominal noise model with Poisson process corrected for FDR with Benjamini-Hochberg procedure.

To further explore these age-related changes in microbiota, we compared aged males to young males (**Figure 4A**). Verrucomicrobia and Tenericutes were significantly more abundant in young male mice than old male mice (**Figure 4A**). Specifically, young male mice had significantly more *Verrucomicrobaceae* and *Ruminococcaceae* than old male mice, while old male mice were enriched with an unclassified family. The first two ordination axes using the PCoA for the weighted ordination across age accounted for 35.5% of sample variation (**Figure 4B**). Additionally, hierarchical clustering partially separated the males by age as well (**Supplementary Figure 3A**). Between young and old male mice, there were 99 significantly different features detected out of 783 tested, with 56 OTUs enriched in old mice while 43 OTUs were enriched in young mice (**Figure 4C**). Specifically, two significant OTUs were identified at the strain level; *Akkermansia muciniphila* was enriched in the young male population (*p=*0.019) while *Clostridium disporicum* was enriched in the old male population (*p=*0.0035). Comparatively, old female mice were then profiled against young female mice (**Figure 4D**). By phyla, old female mice had a significantly higher abundance of TM7 while young female mice had a higher abundance of Tenericutes (**Figure 4D**). Young female mice specifically had a higher abundance of an unclassified family. The first two ordination axes using the PCoA for the weighted ordination across age accounted for 43.3% of sample variation (**Figure 4E**). Hierarchical clustering analysis additionally showed sample separation by age between old and young females (**Supplementary Figure 3B**). Significantly different abundance features were found between young and old females; specifically, there were 157 significantly different features detected out of 800 tested, 97 of these being from old female mice and 60 enriched in young female mice (**Figure 4F**). At the strain level, four significant OTUs were identified: *Streptococcus* sp., *Lactobacillus intestinalis*, and *Lactobacillus reuteri* were enriched in old female mice while *Akkermansia muciniphila* was enriched in young female mice.

**Figure 5:**
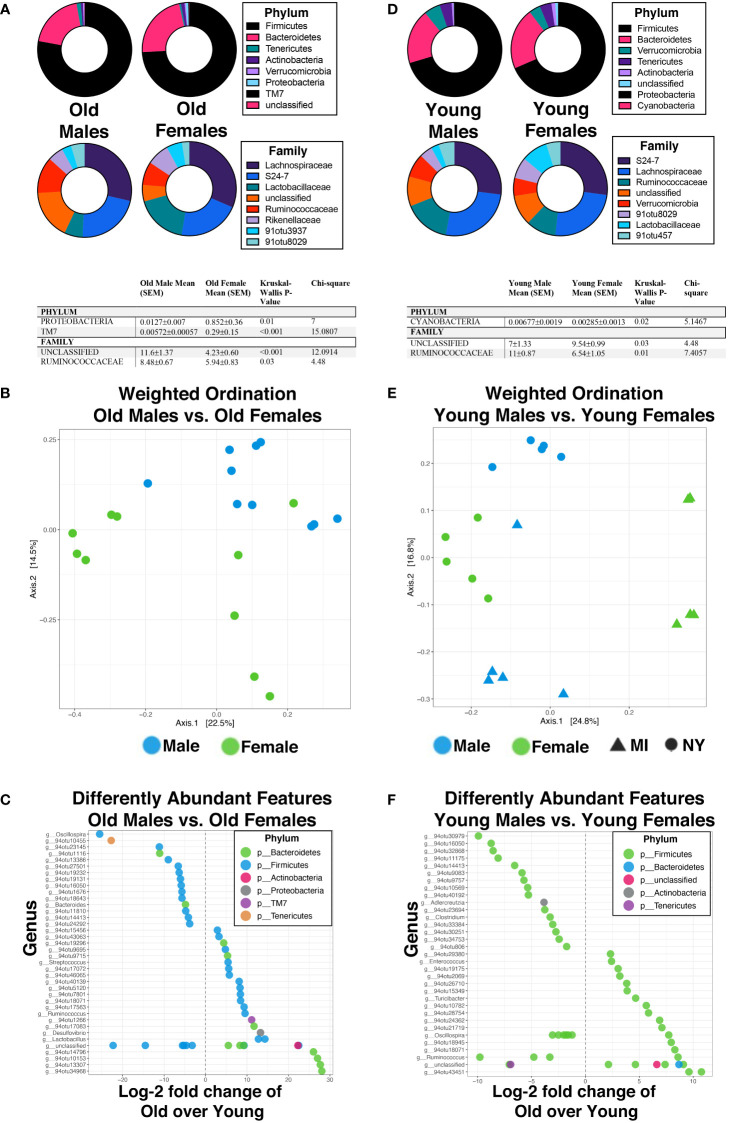
Biological sex directly influences the abundance and diversity of the gut microbiome in an age-independent mechanism in mice. **(A, D)** Percent abundance as means with SEM for the top eight phylum and families expressed between old males and old females **(A)** and young males and young females **(D)**. The two OTUs listed (91otu8029 and 91otu457) belong to the Clostridiales Family. Significant changes (*p*<0.05) are indicated the in the tables below each plot. **(B, E)** Weighted ordination by abundance indicates that samples cluster by age and partially by sex for males **(B)**, and by sex and location for females **(E)**. **(C, F)** Top 50 significantly differently expressed OTUs. Significance was tested using a negative binominal noise model with Poisson process corrected for FDR with Benjamini-Hochberg procedure.

Next, we compared the microbiomes between males and females in the same age groups to better understand the specific differences due to sex. First, we compared all old male mice with old female mice in which we saw significant changes in beta diversity; female mice had significantly higher relative abundance of both Proteobactera and TM7 phylum, though at the family level, significant enrichment was seen in old males in both an unclassified family was well as *Ruminococcaceae* (**Figure 5A**). There was clear separation based on sex when weighted ordination was performed between old males and females (**Figure 5B**). Samples also partially separated by sex based on hierarchical clustering (**Supplementary Figure 4A**). There were 97 significantly different OTUs with 55 enriched in old female mice and 42 enriched in old male mice (**Figure 5C**). Of these, two strain-level OTUs were identified; both *Clostridium saccharogumia* and *Lactobacillus intestinalis* were enriched in old females compared to old males. We then compared the microbiota of young male mice to that of young female mice. Specifically, at the phylum level, Cyanobacteria was significantly more abundant in young male mice (**Figure 5D**) than young female mice. At the family level, *Ruminococcaceae* were increased in young male mice while an unclassified family was increased in young female mice (**Figure 5D**). Despite these changes, weighted ordination using abundance of OTUs showed no separation by sex, but rather partial separation based on location (**Figure 5E**). However, hierarchical clustering by Ward’s method and with Bray-Curtis distance, partial separation by sex between young male and female mice was seen (**Supplementary Figure 4B**). Interestingly, 41 OTUs were enriched in young female mice while 54 were enriched in young male mice (**Figure 5F**); specific changes in strains were seen in an enrichment of *Lactobacillus gasseri* in young females while young males had an enrichment in *Ruminococcaceae bacterium* D16. Together, these data demonstrate changes in the gut microbiota are associated not only with aging but also with sex and somewhat by location. We then asked how the immune system may be involved in the regulation of these changes.

### 3.5 Serum cytokine levels are changed in relation to sex in aging mice

**Figure 6:**
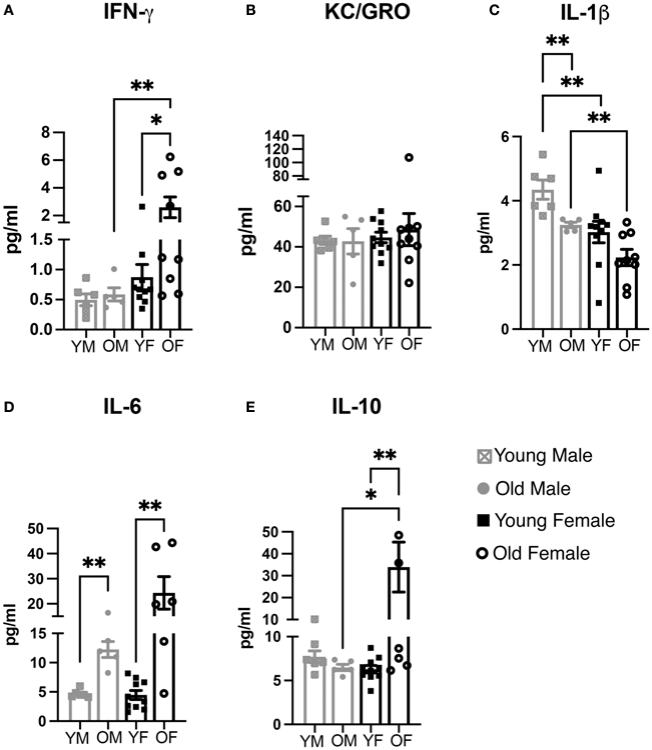
Cytokine biomarkers for inflammation and immune activation are influenced in a sex- and age-dependent mechanism. **(A)** IFN-gamma, **(B)** KC/GRO, **(C)** IL-1beta, **(D)** IL-6, and **(E)** IL-10 were measured in serum obtained from BALB/c-ByJ mice located at WMed, this MI location. Concentrations (in pg/mL) were then averaged (error bars = SEM) for each sex and age group (n=5 old male, n=6 young male, n=9 old female, n=10 young female). Pair-wise comparisons were made using the Mann-Whitney test. Asterisks for *p*-values: *p<0.05, **p<0.01.

Cytokines play a key role in coordinating immune responses including inflammation and pathogen defense (Marín-Aguilar et al; Franceschi et al., 2000; Dabitao et al., 2011; Schirmer et al., 2016). Given the many examples of microbiota modulating immune responses, we investigated the relationship of cytokine responses between these young and old male and female mice. We analyzed several circulating cytokines (IFNγ, IL-10, IL-1β, IL-6, and KC/GRO) from serum collected from young and aged, male, and female mice at the Michigan (WMed) location. We previously published IL-5 serum data from this cohort of mice showing that serum IL-5 is exclusively decreased in old males; old females maintain IL-5 serum levels as compared to young males and females (Webster et al., 2022). We found that cytokine levels exhibited a wide range in each group; average expression levels of IFNγ, IL-10, IL-1β, and IL-6 were found to be significantly differentially expressed between age- and sex-groups while KC/GRO was not (**Figure 6**).

First, we looked at pro-inflammatory cytokines. IFNγ plays an essential role in promotion of the adaptive immune response and immunoregulatory functions (Feng et al., 2021). We found that serum levels of IFNγ changes with both sex and age. Interestingly, IFNγ increases significantly with age in females (0.87pg/mL to 2.6 pg/mL; *p<*0.001). Overall, the level of IFNγ is higher in females compared to males and increases in old females as compared to young females (**Figure 6A**). Next, we examined KC/GRO, which signals through CXCR1 or CXCR2 receptors; in general, KC/GRO proteins chemoattract and activate various monocytes including neutrophils and basophils functioning in inflammation and wound healing (Son et al., 2007). Changes in KC/GRO expression were not significantly different between any age-group nor based on sex (**Figure 6B**). We also examined IL-1β serum levels; IL-1β is considered a trigger for the inflammatory cascade. It functions in a proinflammatory manner causing fever, liver acute-phase response, and even cognitive decline (Banks et al., 2001; Morley and Baumgartner, 2004). Here, we found serum IL-1β levels are highest in young males (4.35pg/mL) while the levels are significantly lower in young females (3.03pg/mL, *p=*0.0047; **Figure 6C**). Interestingly, as mice age, IL-1β levels decrease in both males and females to 3.25pg/mL and 2.23pg/mL, respectively, but this decrease only reaches significance in males (old males vs young males; *p=*0.0043; old females vs young females; *p>*0.05). The expression patterns of IL-1β are the most diverse of the cytokines we examined inasmuch as young males express the highest amounts while old females express the lowest amounts of IL-1β. In addition, we examined expression of serum IL-6. IL-6 is a multi-functional cytokine, and its increase in circulation with age is considered an important marker of acute inflammatory stress (Morley and Baumgartner, 2004). In agreement with previous literature, we found that IL-6 increases with age, but is more significantly increased in females compared to males, a change from 4.686pg/mL to 12.26pg/mL in males (*p=*0.0043) whereas females increased from 4.47pg/mL to 24.34pg/mL (*p=*0.0030; **Figure 6D**). Lastly, we examined the anti-inflammatory cytokine, IL-10. One of the ways IL-10 functions is to inhibit TNFα/IL-1 production (Couper et al., 2008). Expression levels of IL-10 in old females were extremely variable, with levels as high as 87.71pg/mL (**Figure 6E**). On average, young males, young females, and old males express levels between 6.2pg/mL (young female) to 7.72pg/mL (young male) while the average of old females is 33.92pg/mL (*p<*0.05 vs old males; *p<*0.01 vs young females). This large increase unique to old females is interesting as IL-10 has been shown recently to prevent age-associated inflammation (Dagdeviren et al., 2017) and historically has been shown to prevent other age-induced dysfunctions (Lustig et al., 2017).

Taken together, there are significant changes in serum cytokine expression associated with aging in a sex-specific manner; particularly, we found that IFNγ and IL-10 were increased in aged females exclusively, while IL-6 was increased in both aged male and female mice. We were interested to then determine if these age- and sex-specific changes in cytokine expression were associated with the changes in microbial diversity previously observed.

### 3.6 Highly abundant gut microbial species show direct association with serum cytokine levels in an age- and sex-specific manner

**Figure 7:**
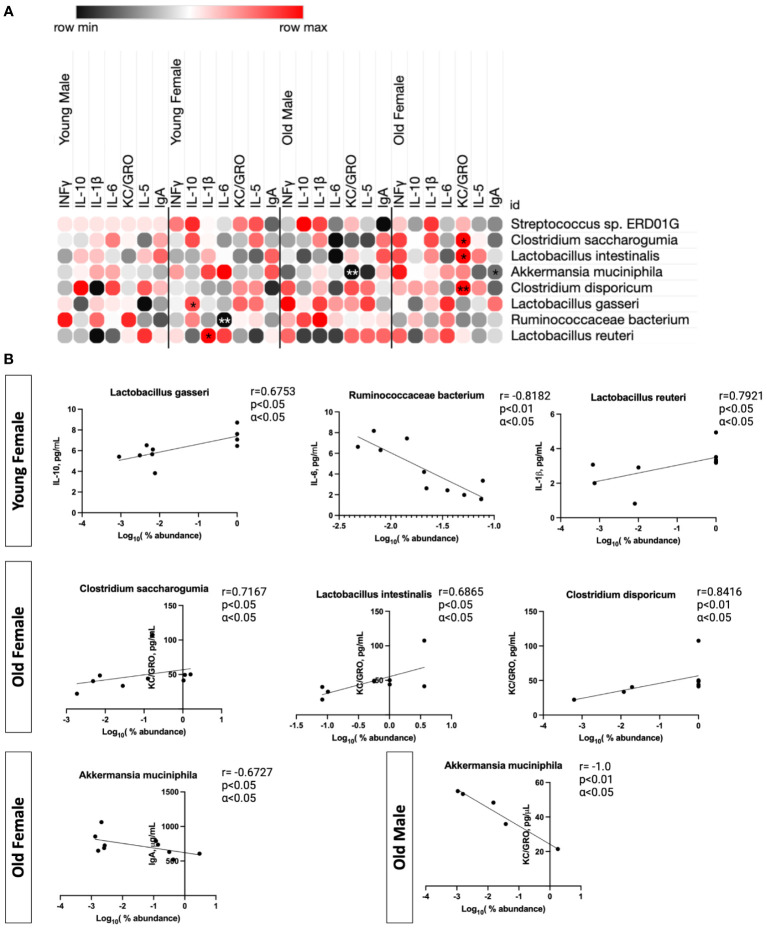
Significant correlations between gut microbial abundances and cytokine response are found in context to aging in a sex-dependent manner. Summary of specific species associations with cytokine responses using Spearman correlation (α <0.05). All species were required to be present in >20% of all samples. Those with positive correlations are shown in red and those with negative correlations are shown in black. **(A)** shows the correlations for percent abundance of each strain and cytokine for each individual group (*p<0.05, **p<0.01). **(B)** Each significant correlation was plotted, and the associated Spearman correlation value (r) was shown in reference to cytokine concentration (pg/mL) and the log_10_(% abundance) of each microbe.

To determine any significant relationships between serum cytokine expression and microbial composition, we performed pairwise correlation tests between microbial taxonomic composition at the strain level that were significantly changed and each cytokine in relation to both age and sex (Spearman correlation with α < 0.05 an a two-tailed *p<*0.05; **Figure 7A**). A total of 19 significant interactions were identified. When young males were analyzed alone, there were no significant correlations with any bacterial strain and cytokine expression. Young females however, had positives correlations between IL-10 and *L. gasseri* (r=0.6753; *p<*0.05), IL-1β and *L. reuteri* (r=0.792; *p<*0.05), and IL-6 was negatively correlated with *R. bacterium* (r= -0.8182; *p<*0.01). These correlations are shown in **Figure 7B**. Old males had only a negative correlation with *A. municipalis* and KC/GRO (r= -1; *p<*0.01; **Figure 7B**) while old females had several significant correlations; *C. saccharogumia*, *L. intestinalis*, and *C. disporcium* were all positively correlated with KC/GRO (**Figure 7B**). In addition, serum IgA was negatively correlated with *A. muciniphila* in old females (r= -0.6727; *p<*0.05).

We then examined correlations between our larger variables singularly; that of sex and that of age. In the young (**Supplementary Figure S5**), the sole significant correlation was a positive correlation between *A. municipalis* and IL-6. However, in the old, several strains were significantly correlated with several cytokines. *Streptococcus* sp. ERD01G was negatively correlated with IFNγ (r = -0.6203; *p<*0.05) and positively correlated with IL-1β (r = 0.7837; *p<*0.01). *C. saccharogumia* was positively correlated with KC/GRO (r = 0.5385; *p<*0.05) as was *C. disporicum* (r = 0.549; *p<*0.05). When sex was examined alone in all males (**Supplementary Figure S6**), IFNγ was positively correlated with *R. bacterium* (r=0.7636; *p<*0.01) and IL-6 was negatively correlated with *A. municipalis* (r=-0.6742; *p<*0.05) in all males. In all females, a correlation was seen in *L. reuteri* negatively with IL-10 (r= -0.6149; *p<*0.01) but positively with IL-1β (r = 0.6628; *p<*0.01). Interestingly, IL-6 was negatively correlated with *Streptococcus* spp. (r= -0.6223; *p<*0.05) and *C. disporcium* was positively correlated with KC/GRO (r= 0.5976; *p<*0.01; **Supplementary Figure S6**).

Together, these data demonstrate that there are important links to the repertoire of serum cytokines and the composition of the microbiome. Importantly, these changes are most noted when looking specifically at the role of sex (male vs female) or age (old vs young). Specifically, we were most interested in IL-10 and IL-6 as they were differentially expressed (**Figure 6**) and displayed significant correlations with bacterial species abundance (**Figure 7** + **Supplementary Figures S5, S6**). As B-1 cells are known to produce IL-6, IL-10, and IgA (Kroese et al., 1989; O’garra et al., 1992; O’garra and Howard, 1992; Kroese and Bos, 1999; Macpherson et al., 2000), which have all been shown to regulate the microbiome, we further explored the role of IL-10 and IL-6 in the signaling of B-1 cells.

### 3.7 IL-10 and IL-6 provide the signal to activate STAT-3 in peritoneal CD5+ B-1 cells

B lymphocytes are involved in the regulation of the gut microbiome through production of IgA (Lycke and Bemark, 2017). CD5+ B-1 cells have been shown to produce approximately half of the gut IgA (Kroese et al., 1989; Duan and Morel, 2006). Interestingly, naïve CD5+ B-1 cells exhibit constitutive activation of STAT3, which is activated downstream of either IL-10 or IL-6 binding their respective receptors. Considering our results demonstrating differences in serum levels of both IL-10 and IL-6, we asked whether these cytokines could contribute to the constitutive activation of STAT3 in CD5+ B-1 cells.

**Figure 8:**
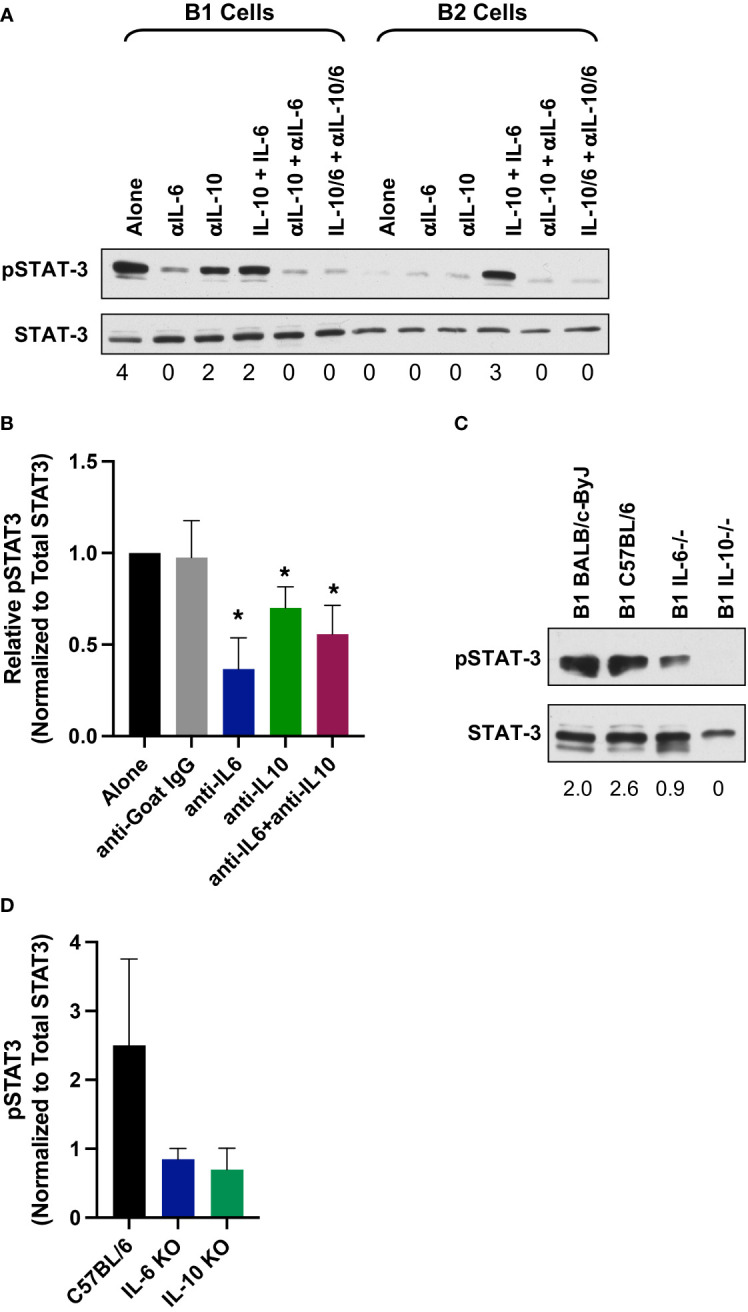
IL-6 and IL-10 provide the signal to constitutive pSTAT3 in CD5+ B-1 cells. **(A)** Sorted CD5+ B-1 and B-2 cells were treated for 24 hours as indicated with IL-10 (10 ng/ml), IL-6 (10 ng/ml), anti-IgG (10 μg/ml), anti-IL10 (10 μg/ml), and/or anti-IL-6 (10 μg/ml). Afterwards, cells were harvested to assess pSTAT3 levels by western blot analysis. Numbers below each band represent relative amount of phospho protein normalized to total STAT3. **(B)** Relative pSTAT3 protein expression normalized to total STAT3; average of four independent experiments. Significance was determined with the Mann-Whitney U test. **(C)** CD5+ B-1 cells were obtained from IL-6 or IL-10 knockout mice and assessed for pSTAT3 by western blot analysis. Numbers below each band represent relative amount of phospho protein normalized to total STAT3. **(D)** Relative pSTAT3 protein expression normalized to total STAT3; average of four independent experiments. Significance was determined with the Mann-Whitney U test (*p<0.05).

Sort purified CD5+ B-1 cells were treated with anti-IL-6 and/or anti-IL-10 neutralizing antibody for 24 hours (**Figure 8**). Afterwards, cells were pelleted, frozen and lysed for western blot analysis of phosphorylated STAT3 (pSTAT3). As a control for the effectiveness of each neutralizing antibody, B2 cells were stimulated with IL-6 and/or IL-10 for 24 hours in the presence or absence of anti-IL-6 and/or anti- IL-10. **Figure 8A** shows B2 cells cultured for 24 hours with IL-6 and IL-10 have phosphorylated STAT3, which is blocked when B2 cells are co-cultured with IL-10, IL-6, anti-IL-10 and anti-IL6. These results demonstrate the effectiveness of the neutralizing IL-6 antibody. Relative pSTAT3 expression from at least four independent experiments including a goat IgG control antibody is presented in **Figure 8B**. Relative pSTAT3 expression was calculated by normalization to total STAT3 by densitometry analysis of scanned gels. Statistical analysis was performed comparing anti-IL-6, anti-IL-10, or anti-IL-6 plus anti-IL-10 to the goat IgG control, which demonstrated all had significantly lowered pSTAT3 protein levels as compared to the IgG control antibody. The pSTAT3 protein levels in the presence of both anti-IL-10 and anti-IL-6 antibodies were not lowered to the same extent as anti-IL-6 alone. The reason for such a discrepancy is unknown. It has been previously shown LIF is involved in the constitutive STAT3 activation observed in CD25 positive CD5+ B-1 cells (Tumang et al., 2011), which could account for the differences seen.

To confirm the IL-6/IL-10 neutralization studies, we examined STAT3 activation in CD5+ B-1 cells obtained from IL-6 and IL-10 knockout mice. **Figure 8C** shows a representative western blot of phospho-STAT3 in CD5+ B-1 cells from IL-6 and IL-10 knockout mice. We found a decrease in phosphorylated STAT3 in both IL-6 and IL-10 knockouts (**Figure 8D**). Together, these results show neutralization or complete lack of IL-6 or IL-10 significantly reduces constitutive pSTAT3 in CD5+ B-1 cells. These results suggest higher serum levels of IL-6 and IL-10 may affect the B-1 CD5+ B-1 cell population, which plays a role in regulation of the microbiome.

## 4 Discussion

There is tremendous interplay between the immune system and the microbes within the body, which makes differentiating the two difficult as both are interdependent for homeostasis. Therefore, the microbiome as an entity could be thought to include both immune components as well as microbes. Studies have shown the influence of intrinsic (age, sex, genetics) and extrinsic (environment, therapeutics) factors upon the immune system and the microbiome in healthy and diseased states; however, these studies have examined such factors independently. Herein, we examine in the same study, how sex and geographical location influence the complete microbiome in context of healthy aging. Our results demonstrate significant differences in the aging-microbiome in a sex-specific manner. Aging accounted for the most change in the microbiome when age, sex, and location were explored, though sex and location played a significant role as well. Healthy aging accounted for large changes in commensal bacteria diversity. Consistent with previous literature, both young and aging mice maintained a high percentage of Firmicutes and Bacteroidetes. However, aging mice lost abundance of Verrucomicrobia and Tenericutes as compared to young mice. Interestingly, abundance of TM7 was increased in healthy aged mice. The loss of TM7 is noteworthy as a more penetrable mucus layer is associated with high prevalence of this bacterial phylum (Jakobsson et al., 2015); additionally, this phylum is also proposed to play a role in intestinal inflammation (Kuehbacher et al., 2008). This data would suggest that healthy aging predisposes the gut to inflammation and a more permeable mucus layer. Supporting this further, we found *Akkermansia muciniphila* was enriched in both young males and young females as compared to aged mice. The loss of *A. muciniphila* with age is consistent with previously published literature (Desai et al., 2016; Greer et al., 2016; Xu et al., 2020); however, this is the first study to our knowledge that determined that age-related loss of *A. muciniphila* is not influenced by other variables such as sex or location. Furthermore, aged mice also had increased abundance of *Lactobacillus intestinalis*, which has been previously associated with metabolic syndrome development (Lecomte et al., 2015; Wang et al., 2020) but, to our knowledge, not previously associated with healthy aging. These data highlight the important changes to the microbiome composition associated with healthy aging in the context of sex.

An interesting finding of this work was the hierarchy of influence between sex, aging and environment. From this study, we found that aging is the most impactful variable on gut microbial composition, followed by sex and then by environment. For example, previous literature found that *L. intestinalis* was increased in very young female mice as compared to very young males (Elderman et al., 2018); however, in the work presented herein, we found that aged females had increased expression of *L. intestinalis* as compared to both aged males but also young females. As such, in our study we were able to specifically compare the changes in diversity associated with sex-specific aging. At the strain level, young males had higher abundance of *A. muciniphila* while aged males were enriched for *Clostridium disporicum*. Similarly, young females were enriched for *A. muciniphila*, while aged females were enriched for *Streptococcus* sp. ERD01G, *Lactobacillus intestinalis*, and *Lactobacillus reuteri*. When males and females were compared in each age group, we found that in aged mice, *Clostridium saccharogumia and L. intestinalis* were enriched in females. In young mice, *Lactobacillus gasseri* was enriched in young females while *Ruminococcacae bacterium* was enriched in young males. The role of biological sex in driving the composition of the microbiome is largely unknown. It has been previously described that genetic background was a stronger determinant of shaping the microbiome composition than sex differences (Kovacs et al., 2011); in the study herein, we found similarly that sex wasn’t the primary driver of microbiome diversity but importantly here, we describe those changes specifically due to sex as they are significant. As we described here, certain changes to the microbiome composition can be attributed to sex-differences primarily, an important factor that previously has been given little attention. Our results clearly demonstrate that sex influences the composition of the aging gut microbiome in the context of age.

Importantly, the environment does play a role in microbiome composition, despite controls used in the laboratory setting. Previous work has demonstrated that the animal facility setting influences the composition of the microbiome due to various factors; however, this study examined young male mice exclusively (Rausch et al., 2016). Here, we examined whether the animal facility influences the composition in relation to aging and biological sex. In the experiments presented here, all mice were housed in comparable AAALAC-approved facilities with identical light-dark cycles, cages, bedding, frequency of cage changes, and comparable food. When all samples were compared, through unweighted ordination and hierarchal clustering, location became a basis for separation. Further, when we examined only the young population by PCoA weighted ordination, the samples separated primarily by location and partially by sex. Further, PERMAOVA microbiome covariate significance identified location as a variable that significantly contributed to the beta diversity of samples. These data raise the unexpected possibility of mouse microbiome differences reflecting fomites from human handlers. As the microbiome can influence immune parameters, this adds another dimension to the issue with respect to reproducibility and universality of experimental results from any given laboratory. Previously, it has been recognized that different genetic strains may be manifesting different immune parameters and responses (Petkova et al., 2008); herein we show location of experimentation should be considered in addition to age and sex in terms of particulars that may influence findings in mice. Such differences in bacterial strains present in the mouse microbiota has been previously appreciated in mice obtained from different vendors, which affected the frequency of Th17 cells (Ivanov et al., 2009).

It has been well established that diet impacts the composition and function of the microbiome [reviewed in (Ruan et al., 2020)]. Recently, more attention has been drawn to the role of diet in the reproducibility of microbiome work in the laboratory (Tuck et al., 2020). Slight changes were seen in the microbiome with varying diets however, the specific elements within the diets causing the changes in microbiota composition could not be elucidated. Here, we examined the role of two different animal housing locations in context to aging and sex in microbiome composition. We found location does play a role in microbiome diversity but that it is tertiary to that of age and sex. However, our experimental paradigm while nearly identical in housing varies in a few ways: 1) the human handlers for the animal rooms, 2) the composition of the deionized water, and 3) the small difference in diet. As compared to FMIR, mice at WMed were fed diets that consisted of additionally ground soybean hulls and casein (**Supplementary Table S1**). Interestingly, work has shown that both soy and bovine products can influence the gut microbiome when fed a high-fat diet (Ijaz et al., 2018). However, it is unclear if these specific dietary changes can influence the gut microbiome in context to aging in both males and females. Additionally, as the specific chemical content of diet differs lot to lot, further work exploring these variables is warranted.

**Figure 9:**
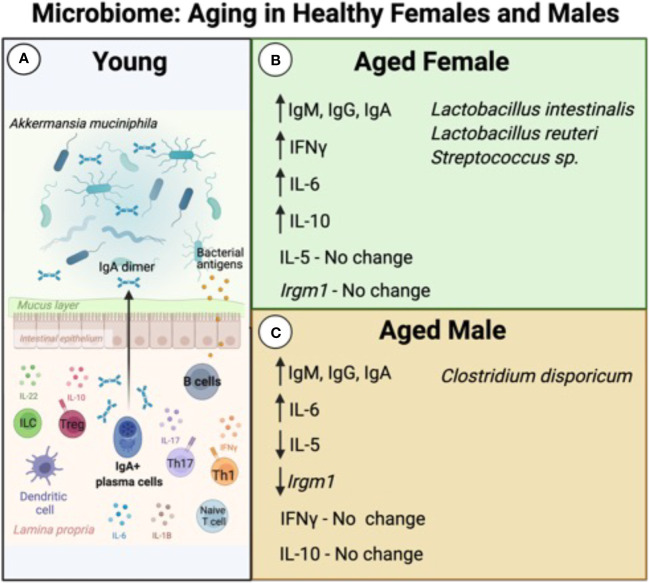
Summary of Microbiome and Immune System Aging in Healthy Females and Males. The composition of the microbiome of young mice **(A)** is maintained by a complex interplay of both adaptive and innate immune cells and cytokines. IgA secreted by B lymphocytes is important for maintenance of beneficial bacteria, such as *Akkermansia muciniphila*. During the aging process, the microbiome is influenced in a sex-dependent manner. **(B)** Aged females have increased expression of IgM, IgG, and IgA, as well as increased serum levels of IFNγ, IL-6, and IL-10. There is no change in expression of IL-5 as compared to young animals. These changes are correlated with increased abundance of *L. intestinalis*, *L. reuteri*, and *Streptococcus* sp. **(C)** Aged males have increased production of IgM, IgG, and IgA as well as IL-6 but have decreased expression of IL-5. There are no changes in serum expression levels of IFNγ nor in IL-10. These changes in expression are correlated with increased abundance of *Clostridium disporicum*. Figure was created with BioRender.com.

The microbiome plays critical roles in the development of the immune system while the immune system maintains key features of host-microbe homeostasis (Zheng et al., 2020) yet, the effects of sex during the aging process in these relationships are not well understood. Herein, we found serum cytokine levels to be differentially expressed between aged females and males. Aging has been shown to significantly change the landscape of cytokine production; some biomarkers of aging include increased levels in IL-6, which leads to bone and muscle loss, fever, activation of hepatic acute phase response, and decline in haemoglobin levels (Morley and Baumgartner, 2004). In addition, the anti-inflammatory cytokine IL-10 produced by both innate and lymphoid cells, has been reported to increase in elderly people (Rea et al., 2018). Herein, we examine these and other cytokines; we found that aged females have elevated levels of serum IL-6, IL-10, and IFNγ as compared to young females whereas aged males have elevated levels of IL-6 as compared to young males. **Figure 9** summarizes these findings. Since cytokines have been shown to play a role in the microbiome (Morhardt et al., 2019; Guo et al., 2020; Liu et al., 2021; Marie et al., 2021), the changes in cytokine levels we observed with age and sex could impact microbiome homeostasis.

Examining the interactions between the serum cytokine levels and abundance of specific strains, we found that there were several significant correlations. The increase in IL-6 during the aging process is interesting in context to the microbiome. Previous literature has shown that IL-6 is required for a robust mucosal barrier and effective communication between gut epithelial cells and immune cells [reviewed in (Guo et al., 2020)]. Here, we found that IL-6 is positively correlated with *A. muciniphila* in young mice. IL-10 has previously been shown to be produced by B cells in the gut to maintain specific microbial components (Mishima et al., 2019). Herein, we found IL-10 to be positively correlated with *L. gasseri* in young females. Based on the analysis done in this study, there are significant correlations between serum cytokine profile and maintenance of abundance of commensal bacteria. Sex hormones have also been shown to modulate the expression of cytokines, though there are large gaps in our understanding of the specifics of this modulation. For example, testosterone, which declines in aging males, suppresses IL-6 and IL-1β in humans (Mohamad et al., 2019). Additionally, it has been shown in both mice and humans, that IL-2 and IFNγ are produced by lymphocytes stimulated with dehydroepiandrosterone sulphate (DHEA-S) (Daynes et al., 1990; Suzuki et al., 1991) further indicating sex hormones may influence the active state of the immune system. What is unclear is whether cytokine production is due to the direct activation of immune cells by hormones, or rather by an indirect/secondary effect. Commensal bacteria have also been shown to provide an alternate source of hormones [reviewed in (Clarke et al., 2014)]. These previous studies and the results herein demonstrate the integral connections between microbiota, immune mediators, endocrine mediators, and age.

CD5+ B-1 cells can secrete both IL-6 and IL-10 (O’garra et al., 1992; O’garra and Howard, 1992; Margry et al., 2014). Here, we demonstrate that IL-6 and IL-10 are, in part, responsible for the constitutive activation of STAT3 seen in CD5+ B-1 cells, which has also been shown to be downstream of LIF signalling (Tumang et al., 2011). CD5+ B-1 cells play a role in regulation of the microbiome as they produce a significant proportion of mucosal IgA (Kroese et al., 1989; Cebra et al., 1994; Kroese et al., 1996; Snider et al., 1999). It is currently unknown how altered cytokine levels and/or microbial diversity may affect mucosal CD5+ B-1 cells and their role in microbiome homeostasis; however, we recently demonstrated peritoneal CD5+ B-1 cells display sex-specific differential expression of *Irgm1* in the aged (Webster et al., 2022). Interestingly, the IFNγ-modulating gene *Irgm1* has been identified as regulating the abundance of *A. muciniphila*, where higher levels of *Irgm1* expression are shown to downregulate the activity and abundance of these bacteria (Greer et al., 2016; Azzam et al., 2017). Recent work has demonstrated that *A. muciniphila constitutes* 3-5% of the healthy adult human microbiome, but the levels vary according to many factors including aging; reduced abundance of *A. muciniphila* is one of the first hallmarks of a dysregulated microbiome (Zhang et al., 2015; Greer et al., 2016; Bodogai et al., 2018; Xu et al., 2020). Our results demonstrate that the aging process results in the loss of *A. muciniphila* and this is not sex dependent. Interestingly, IFNγ can increase expression of *Irgm1* in a negative feedback loop (King et al., 2011) and loss of *Irgm1* has been shown to induce increased cytokine production and increased intestinal inflammation (Liu et al., 2013; Taylor et al., 2020). We previously demonstrated CD5+ B-1 cells obtained from aged females show no change in *Irgm1* expression (Webster et al., 2022). Taken together, our data suggest that the loss of *A. muciniphila* we observe in aged mice is not due to *Irgm1*-specific downregulation by CD5+ B-1 cells. Yet, we observe increased IL-6 expression in both male and female mice, which could lead to an over production of IgA (Ramsay et al., 1994) and in turn, possibly negatively regulate the percent abundance of beneficial bacteria, such as *A. municinipila*. Interestingly, we have observed a significant increase in serum IgA levels in aged males and females (Webster et al., 2022) and importantly, the increased levels of IgA in females does significantly correlate with the loss of *A. muciniphila* (**Figure 7**). However, it remains unknown if B-1 B cells are influencing the composition of the microbiome in this sex-specific manner or if the microbiome is regulating these developmental changes associated with aging B-1 cells. Further work is required to better understand this important relationship.

Our results demonstrate that the composition of the microbiome is associated with changes in age, sex, and housing environment of mice. Furthermore, we show aged mice had higher relative abundance of TM7 species and lower relative abundance of Verrucomicrobia and Tenericutes than the young mice. Further, regardless of sex, aged mice had lower relative abundance of *A. municiphila*, an important microbe known to be associated with gut health (Xu et al., 2020). In support of these findings, a recent publication in C57BL/6J males across lifespan was also shown to have an age-associated loss of *A. municiphila* (Low et al., 2022). Our results demonstrate many differences in the microbiome were also associated sex, with more pronounced sex-specific differences in the aged population. Interestingly, when sex-specific differences are examined in the young population, they are present but influenced more by the location in which the animals were raised. We conclude that aging mice undergo sex-associated alterations in the gut microbiome with a distinct serum cytokine profile.

Immunological profiles are distinct in males and females; increased expression of IL-6 was seen in aged mice while increased expression of IL-10 was seen in aged female mice exclusively. We further demonstrated that IL-10 and IL-6 activate STAT-3 in CD5+ B-1 cells, which play a role in microbiome maintenance through IgA production (Kroese et al., 1989; Cebra et al., 1994; Kroese et al., 1996; Snider et al., 1999). Importantly, differentially abundant microbes in aged animals show direct associations with serum cytokine levels including IL-1β, IL-10, IL-6, IFNγ, and KC/GRO as seen with correlation analysis. In conclusion, both age and sex play a significant role in the relationship between the immune system and the microbiome.

## Data availability statement

The data presented in the study are deposited in the NCBI BioSample repository, accession numbers: SAMN26262922: BALB-F-FIMR-O1-R1, SAMN26262923: BALB-F-FIMR-Y1-R1, SAMN26262924: BALB-F-WMED-A1-R1, SAMN26262925: BALB-F-WMED-Y1-R1, SAMN26262926: BALB-M-FIMR-O1-R1, SAMN26262927: BALB-M-FIMR-Y1-R1, SAMN26262928: BALB-M-WMED-A1-R1, SAMN26262929: BALB-M-WMED-Y1-R1.

## Ethics statement

The animal study was reviewed and approved by institutional animal care and use committee at the Feinstein Institutes for Medical Research and the Institutional Animal Care and Use Committee at Western Michigan University Homer Stryker M.D. School of Medicine.

## Author contributions

SW performed the experiments, analyzed data, interpreted the data, and was a major contributor to writing the manuscript. DV analyzed and interpreted the data. TR analyzed and interpreted the data and edited the manuscript. NH performed experiments, analyzed data, interpreted the data, and edited the manuscript. All authors contributed to the article and approved the submitted version.

## Funding

Funding was provided by the National Institute of Allergy and Infectious Diseases of the NIH under R01AI154539 (NH) and NIH AI029690 (TR). The content is solely the responsibility of the authors and does not necessarily represent the official views of the National Institutes of Health.

## Acknowledgments

A sincere thank you to Mireya del Carmen Diaz Insua and Michael Clemente for thoughtful and helpful discussions on data analysis and manuscript preparation. We also acknowledge the outstanding technical support from the Flow Cytometry and Imaging Core at WMU Homer Stryker M.D. School of Medicine.

## Conflict of interest

The authors declare that the research was conducted in the absence of any commercial or financial relationships that could be construed as a potential conflict of interest.

## Publisher’s note

All claims expressed in this article are solely those of the authors and do not necessarily represent those of their affiliated organizations, or those of the publisher, the editors and the reviewers. Any product that may be evaluated in this article, or claim that may be made by its manufacturer, is not guaranteed or endorsed by the publisher.
