# Supplementary material for: Modulation of microbiome diversity and cytokine expression is influenced in a sex-dependent manner during aging
Source: Front Microbiomes. 2022 Oct 10;1:994464. doi: 10.3389/frmbi.2022.994464 (PMC10328149; doi:10.3389/frmbi.2022.994464)
Supplement: Supplementary file 1 [file DataSheet_1.pdf]

1 Supplementary Figures

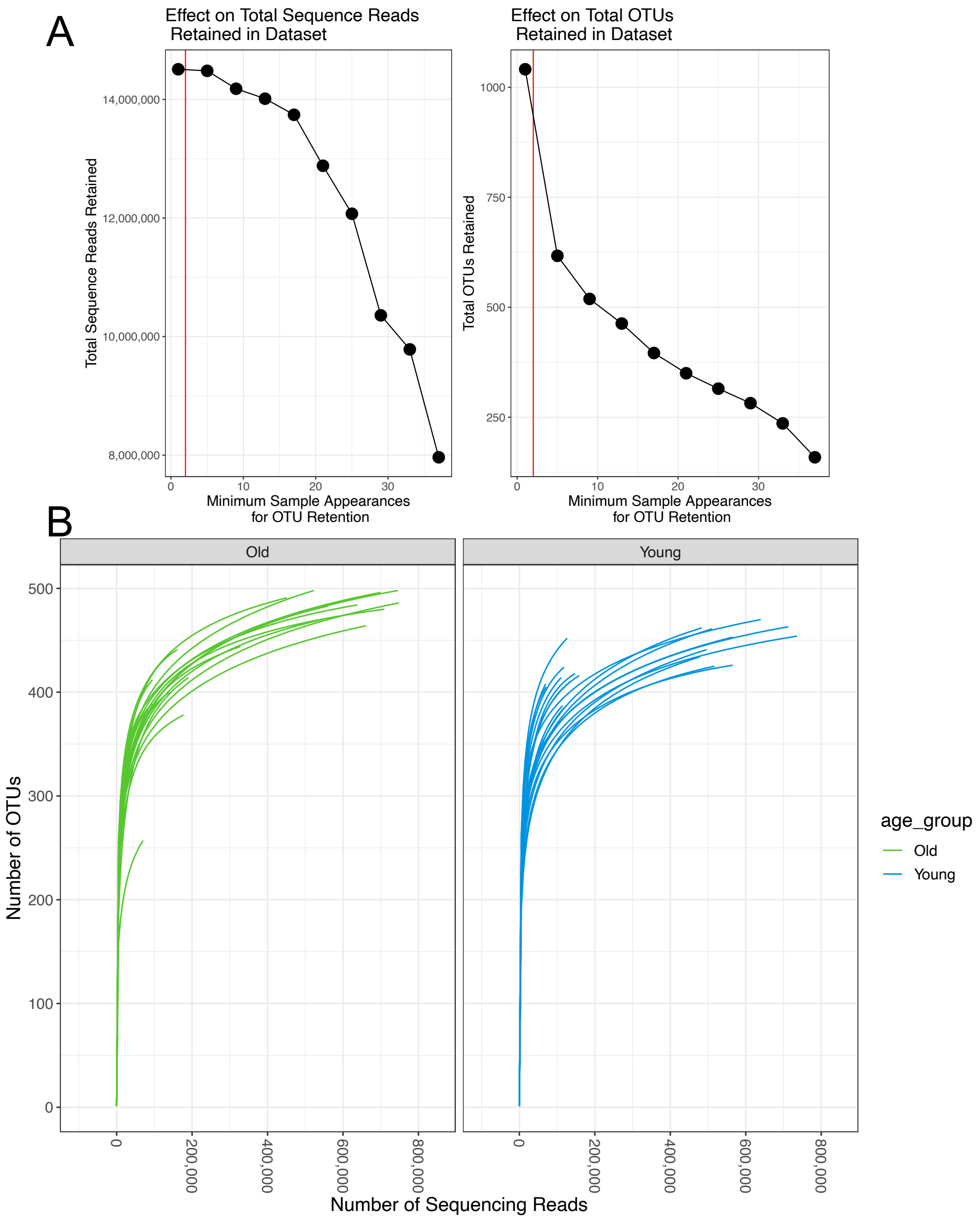

Supplemental Figure S1. Generation of 16S rRNA Analysis. (A) Removal of spurious OTUs were completed by independent filtering. OTUs seen at least once within 5% of the dataset were kept. Filtering reduced the number of OTUs from 969 to 935, and the number of sequences from 13,915,629 to 13,915,585. (B) Rarefaction curve analysis of average number of OTUs detected vs sequencing library size. Rarefaction curves for all samples approached saturation indicating that communities were sufficiently sampled to characterize the microbiome.

A Sequencing Summary

Parameter	Value
Number of samples	40
Total reads	39,808,118
Total reads paired	12,728,890
Reads with strain hits	292,533
Reads without strain hits	12,436,357
Total quality-filtered reads	12,345,288
Total reads matched to raw OTUs	12,231,930
Total reads matched to filtered OTUs	12,231,644
Average read length	251
Average length of paired reads	271
Average read quality	35
Average read quality of paired reads	38
Total strain-hit OTUs	148
Total de novo raw OTUs	1,237
Total de novo filtered OTUs	1,094
Combined number of OTUs	1,242
Combined number of sequences	12,524,177
Number of sequences after removal of OTUs unclassified at kingdom level	14,509,060
Number of OTUs after removal of OTUs unclassified at kingdom level	1,136

B Sequencing Classification by Taxonomic Rank

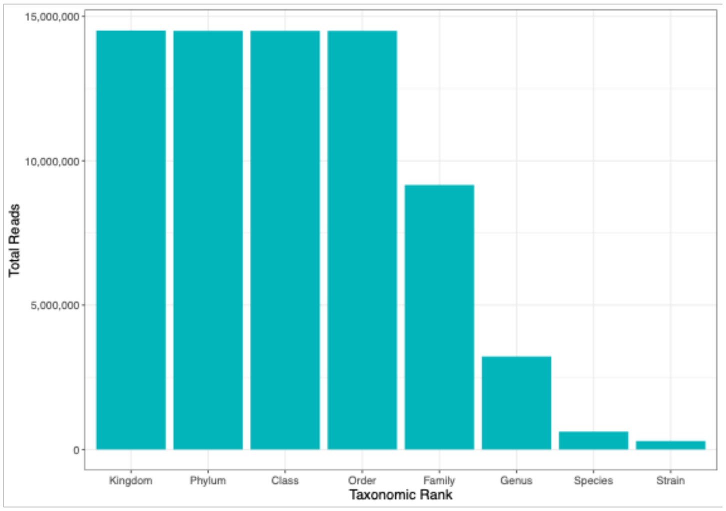

C Unweighted Ordination

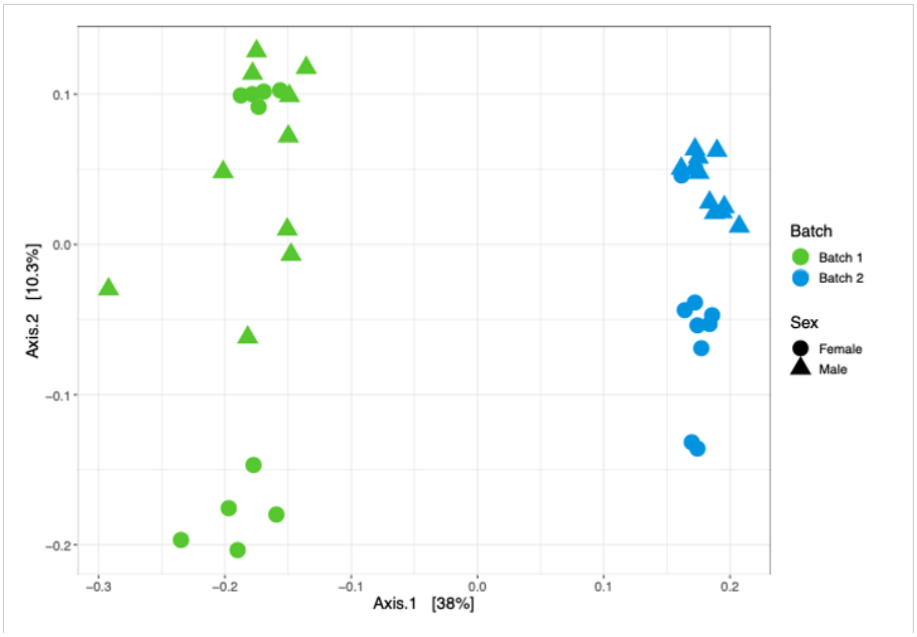

Supplemental Figure S2. 16S rRNA Sequence Analysis. (A) Sequencing summary statistics from the Veracet pipeline. Numbers may include positive and negative sequencing controls, which are excluded in further analysis. (B) Summary of taxonomic classification success of sequences for all samples. Kingdom, phylum, class, and order were all classified at 99.9% or higher. (C) Unweighted ordination by the dimensional reduction of the Jaccard distance between microbiome samples using the PCoA ordination method shows samples separating by location (p=0.001; PERMANOVA), rather than age or sex. Here, blue is representative of WMed and green is FMIR. Females are circles and males are triangles.

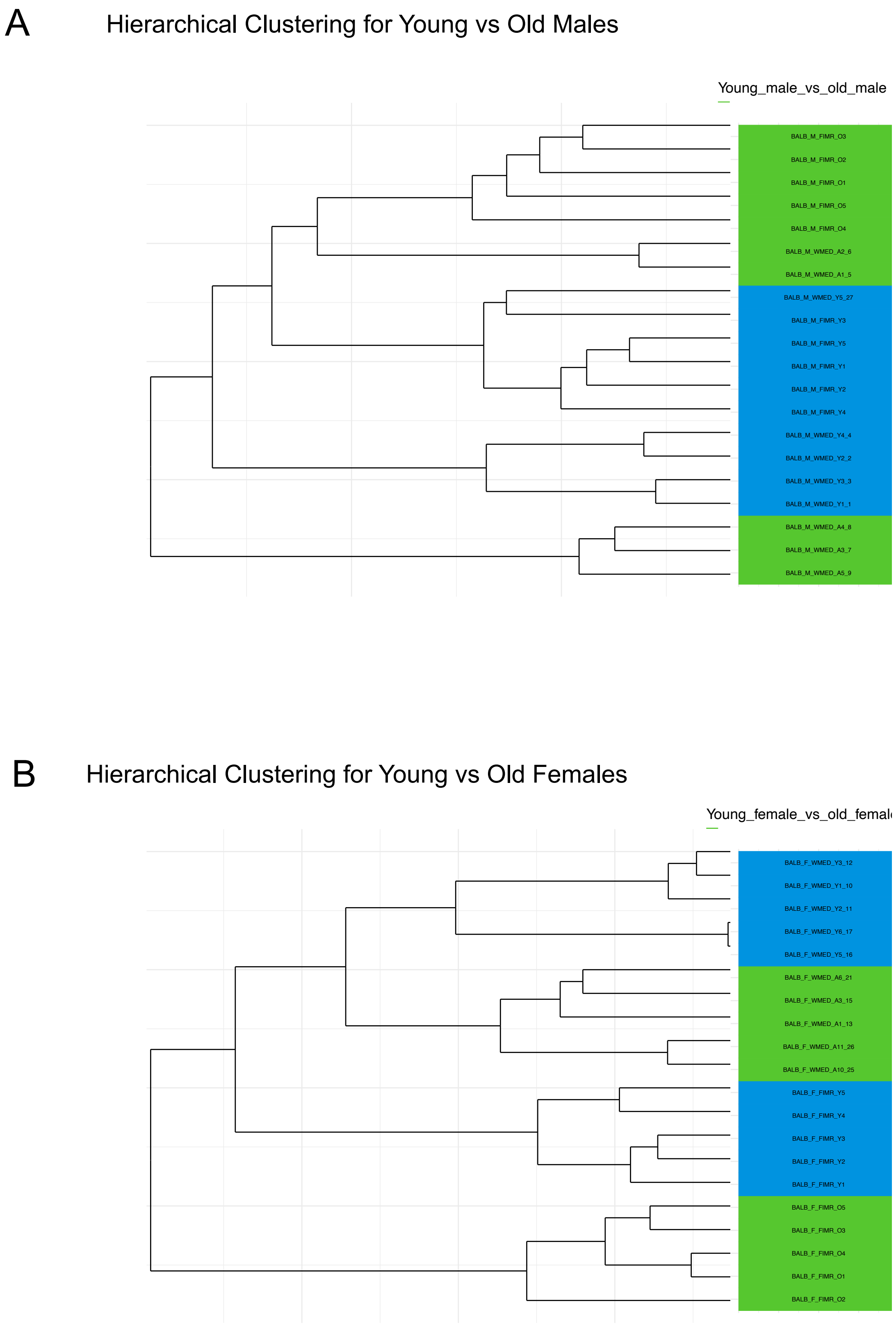

Supplemental Figure S3. Hierarchal Clustering of Microbiome Samples by Age. Samples were clustered by the Ward's method with Bray-Curtis distance for (A) young vs old males and (B) young vs old females. Green indicates an aged sample while blue indicates a young sample.

A Hierarchical Clustering for Old Males vs Old Females

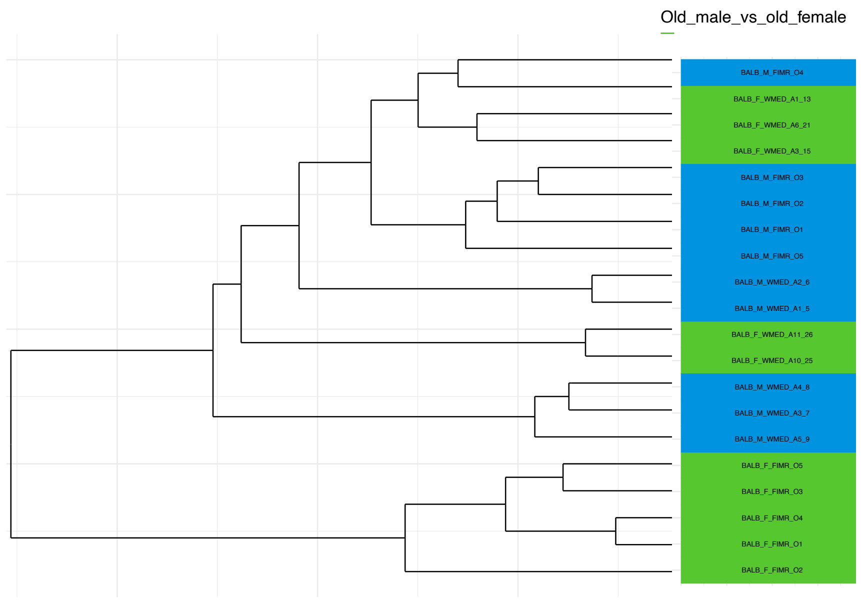

B Hierarchical Clustering for Young Males vs Young Females

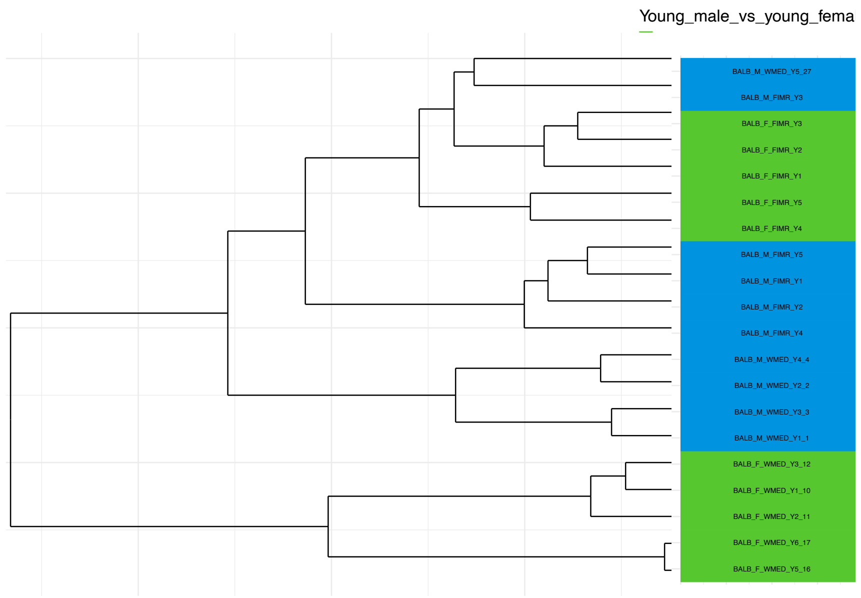

Supplemental Figure S4. Hierarchal Clustering of Microbiome Samples by Sex. Samples were clustered by the Ward's method with Bray-Curtis distance for (A) old females vs old males and (B) young males vs young females. Here, blue represents male samples while green represents females.

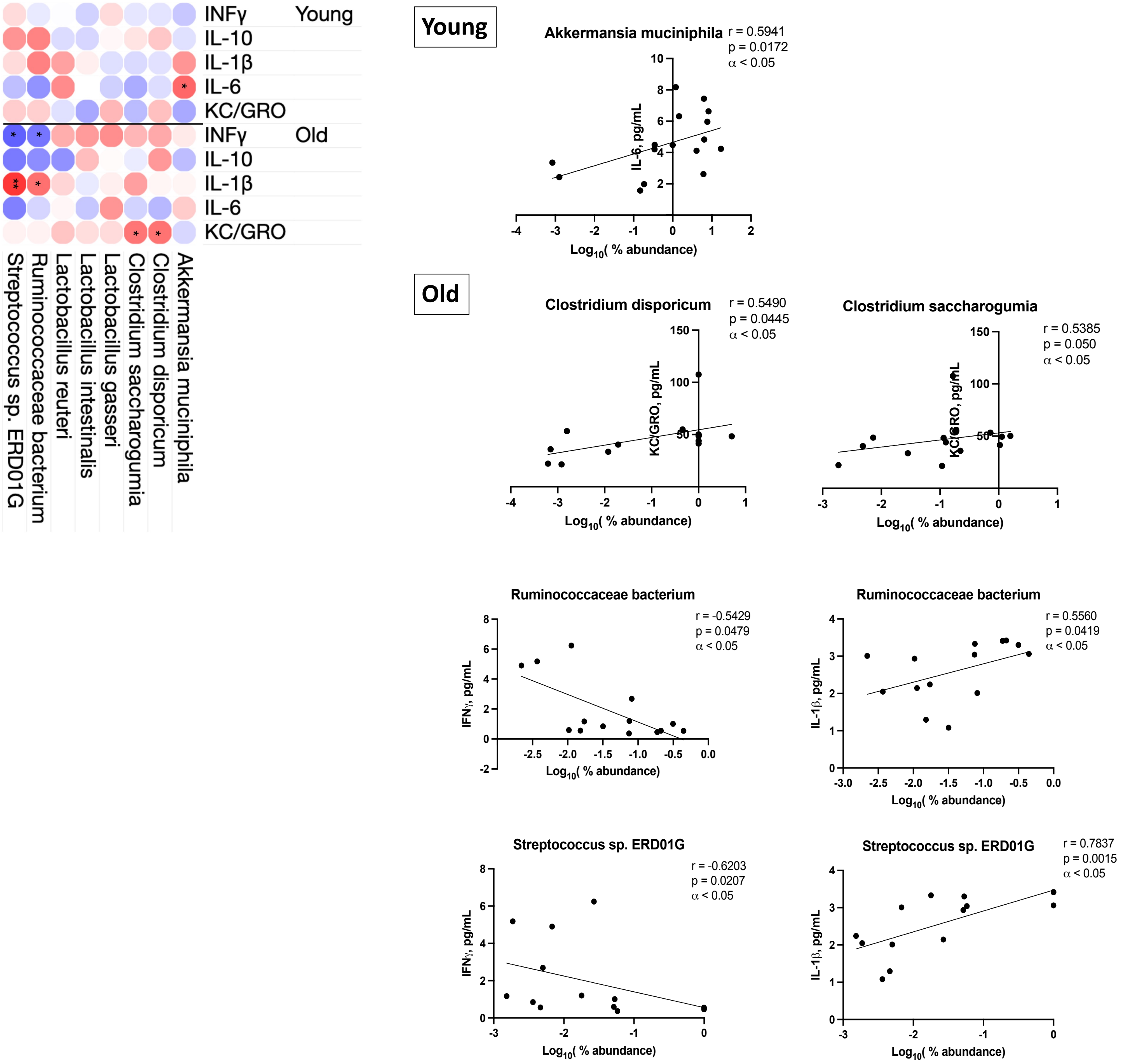

Supplemental Figure S5. Age-specific correlations between gut microbial abundances and cytokine responses. Summary of specific species associations with cytokine responses using Spearman correlation (α <0.05). All species were required to be present in >20% of all samples. Those which positive correlations are shown in red and those with negative correlations are shown in blue. (A) shows the correlations for percent abundance of each strain and cytokine for each individual group (* p<0.05, ** p<0.01). (B) each significant correlation was plotted, and the associated Spearman correlation value (r) was shown in reference to cytokine concentration (pg/mL) and the $\log_{10}$(% abundance) of each microbe.

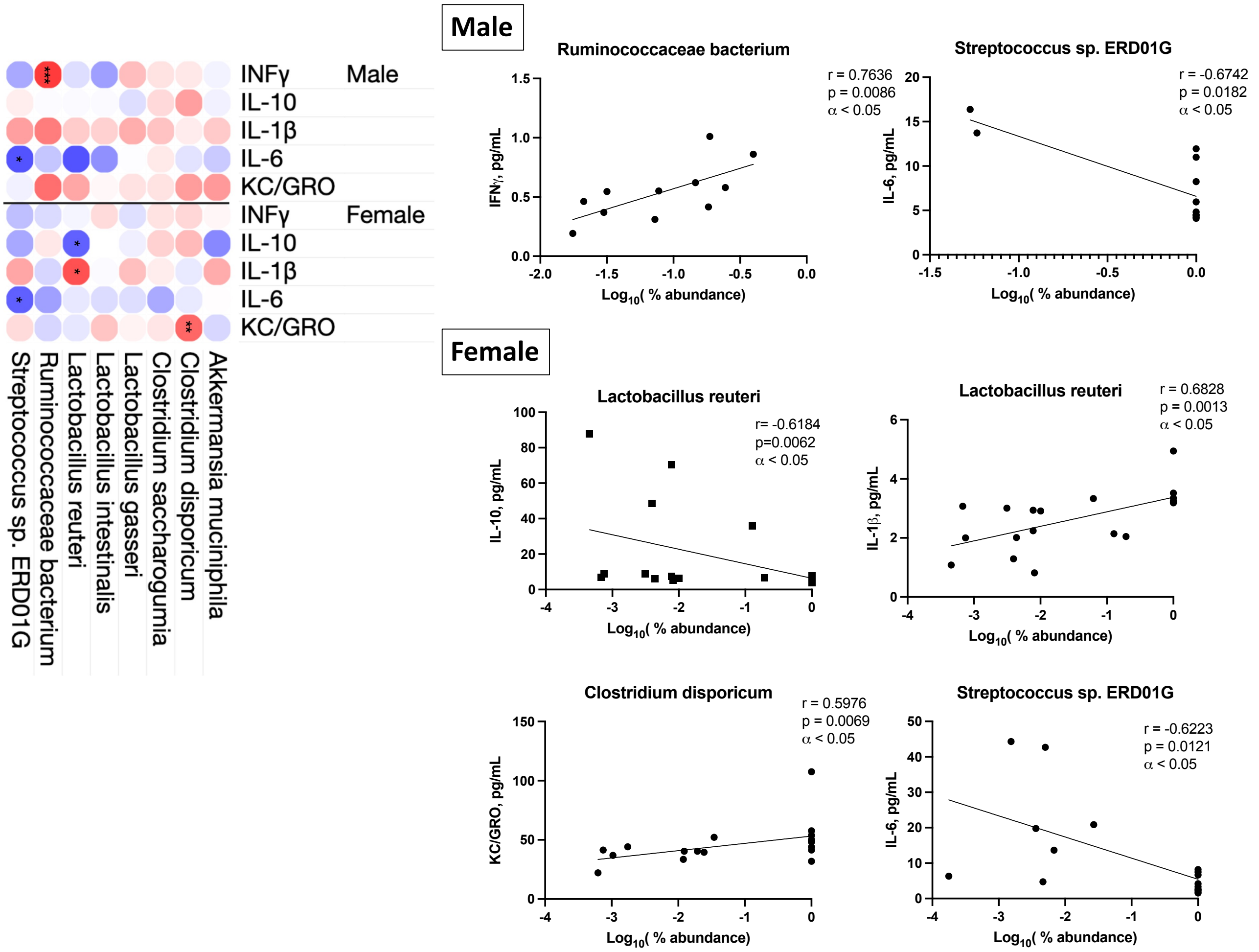

Supplemental Figure S6. Sex-specific correlations between gut microbial abundances and cytokine responses. Summary of specific species associations with cytokine responses using Spearman correlation (α <0.05). All species were required to be present in >20% of all samples. Those which positive correlations are shown in red and those with negative correlations are shown in blue. (A) shows the correlations for percent abundance of each strain and cytokine for each individual group (* p<0.05, ** p<0.01, *** p<0.001). (B) each significant correlation was plotted, and the associated Spearman correlation value (r) was shown in reference to cytokine concentration (pg/mL) and the $\log_{10}$(% abundance) of each microbe.

Table S1. Full ingredient list and macronutrient comparison of laboratory mouse chow

Component	5053	5002
Nutrients		
Protein %	21.0	20.7
Arginine %	1.29	1.22
Cysteine %	0.36	0.35
Glycine %	0.98	0.92
Histidine %	0.53	0.52
Isoleucine %	0.87	0.87
Leucine %	1.58	1.59
Lysine %	1.18	1.19
Methionine %	0.62	0.43
Phenylalanine %	0.92	0.90
Tyrosine %	0.61	0.62
Threonine %	0.79	0.79
Tryptophan %	0.24	0.23
Valine %	0.97	0.97
Serine %	1.00	0.98
Aspartic Acid %	2.23	2.20
Glutamic Acid %	4.26	4.16
Alanine %	1.20	1.22
Proline %	1.32	1.35
Taurine %	0.03	0.03
Fat (ether extract) %	5.0	5.0
Fat (acid hydrolysis) %	6.3	6.3
Cholesterol ppm	135	142
Linoleic Acid %	2.32	2.11
Linolenic Acid	0.28	0.26
Arachidonic Acid %	0.02	0.01
Omega-3 Fatty Acids %	0.42	0.42
Total Saturated Fatty Acids %	0.77	0.92
Total Monounsaturated Fatty Acids %	1.00	0.98
Fiber (crude) %	4.4	4.6
Neutral Detergent Fiber %	15.5	15.5
Acid Detergent Fiber %	5.6	6.0
Nitrogen-Free Extract (by difference) %	53.5	52.8
Starch %	28.2	30.6
Sucrose %	2.71	3.22
Total Digestible Nutrients %	75.1	75.6
Gross Energy kcal/ gm	4.11	4.11
Physiological Fuel Value kcal/gm	3.43	3.43
Magnesium %	**0.21**	**0.21**
Sulfur %	0.31	0.27
Sodium %	0.30	0.30
Chloride %	0.53	0.53
Fluorine ppm	9.2	9.9
Iron ppm	184	190
Zinc ppm	79	83
Manganese ppm	82	76
Copper ppm	13	13
Cobalt ppm	0.72	0.76
Iodine ppm	0.97	0.98
Chromium (added) ppm	0.01	0.01
Selenium ppm	0.37	0.34
Vitamins		
Carotene ppm	1.5	1.3
Vitamin K ppm	3.3	1.3
Thiamin ppm	16	15
Riboflavin ppm	8.1	8.1
Niacin ppm	84	87
Pantothenic Acid ppm	17	17
Choline ppm	1575	1580
Folic Acid ppm	3.0	3.1
Pyridoxine ppm	9.6	6.0
Biotin ppm	0.30	0.30
B12 mcg/kg	51	51
Vitamin A IU/gm	15	15
Vitamin D (added) IU/gm	2.3	2.3
Vitamin E IU/kg	99	65
Ascorbic Acid mg/gm	0.00	0.00

Ingredients (5053): ground corn, dehulled soybean meal, wheat middlings, ground wheat, fish meal, dried plain beet pulp, cane molasses, wheat germ, brewers dried yeast, ground oats, dehydrated alfalfa meal, soybean oil, dried whey, calcium carbonate, salt, DL-Methionine, Menadione, Dimethylpyrimidinol bisulfite (vitamin K), choline chloride, pyridoxine hydrochloride, cholecalciferol (vitamin D3), vitamin A acetate, DL-alpha tocopheryl acetate (vitamin E), folic acid, thiamine mononitrate, manganous oxide, vitamin B12 supplement, zinc oxide, ferrous carbonate, nicotinic acid, riboflavin supplement, calcium pantothenate, copper sulfate, zinc sulfate, calcium iodate, cobalt carbonate, biotin, sodium selenite

Ingredients (5002): ground corn, dehulled soybean meal, ground wheat, wheat middlings, fish meal, wheat germ, dried plain beet pulp, brewers dried yeast, cane molasses, ground oats, soybean oil, dehydrated alfalfa meal, ***ground soybean hulls***, dried whey, ***casein***, calcium carbonate, salt, choline chloride, DL-Methionine, Menadione, dimethylpyrimidinol bisulfate (vitamin K), cholecalciferol (vitamin D3), vitamin A acetate, pyridoxine hydrochloride, DL-alpha tocopheryl acetate (vitamin E), folic acid, dicalcium phosphate, thiamine mononitrate, manganous oxide, vitamin B12 supplement, zinc oxide, ferrous carbonate, nicotinic acid, calcium pantothenate, riboflavin supplement, copper sulfate, zinc sulfate, calcium iodate, cobalt carbonate, biotin, sodium selenite

**Ingredients that are not found in both chows are highlighted in bold italic*

Table S2. Alpha Diversity Measures

Sex	Age	OTU	Shannon	*p*-value (KW)
All Females		434±65.3	3.97±0.475	OTU: p=0.11<br>Shannon: p=0.88
All Males		430±25.6	4.17±0.24	
Male	Old	417±84.4	4.19±0.19	OTU: p=0.94<br>Shannon: p=0.94
Male	Young	435±28.9	4.15±0.29	
Female	Old	452±35	3.93±0.49	OTU: p=0.11<br>Shannon: p=0.88
Female	Young	426±22.3	4±0.47	
